# Nightly torpor use in response to weather conditions and individual state in an insectivorous bat

**DOI:** 10.1007/s00442-021-05022-6

**Published:** 2021-08-28

**Authors:** Mari Aas Fjelldal, Jonathan Wright, Clare Stawski

**Affiliations:** Department of Biology, Norwegian University of Science and Technology (NTNU), 7491 Trondheim, Norway

**Keywords:** Energy budget, Heterothermy, Lunar phobia, *Nyctophilus bifax*, Radio telemetry

## Abstract

Torpor is a well-known energy conservation strategy in many mammal and bird species. It is often employed when environmental conditions are unfavourable to maximize survival probabilities. However, torpor often carries with it the physiological costs of a low body temperature and of rewarming in addition to potential missed opportunities for foraging. Therefore, we hypothesised that decision making regarding when to use torpor should reflect the most important environmental conditions for species distributions, and thus how they may be impacted by ongoing climate change. We investigated how weather conditions affect nightly torpor patterns in the nocturnal insectivorous Australian eastern long-eared bat (*Nyctophilus bifax*). By measuring the skin temperature of 37 free-ranging individuals, we confirmed that torpor was used more frequently during the winter and at subtropical compared to tropical locations. Using mixed-effect models we show that lower ambient temperatures were the main driver of individual torpor use, probably due to lower roost temperatures and prey availability. However, increased rain, wind and humidity, and decreasing barometric pressure, as well as brighter moonlight, also led to more time spent torpid per night. We suggest that bats evaluate multiple environmental cues to make decisions regarding torpor use versus active foraging based upon their expectations of the energetic benefits, prey availability and relative predation risk. Interactions between some of these effects and body mass (whilst controlling for forearm length) indicate that individual variation in body size and/or state-dependent effects of energy reserves also partly determined the use of nightly torpor in these bats.

**Supplementary Information:**

The online version contains supplementary material available at 10.1007/s00442-021-05022-6.

## Introduction

Seasonality and daily variation in weather can inflict substantial energetic costs on endotherms that have to constantly balance their energy budget to maintain a stable body temperature (*T*_b_). Increases of thermoregulatory and body maintenance costs during poor weather conditions result in animals needing to compensate for the energy lost, for example through increased foraging rates. However, many food sources are also seasonal or vary with weather conditions, which for many species can lead to a mismatch between energy requirements and resource availability. In a range of endotherm species we, therefore, find strategies such as daily torpor and hibernation (temporal heterothermy) that are characterized by reductions in metabolic processes and a decrease in *T*_b_ (Ruf and Geiser 2015). The energy requirements of torpid animals are thus greatly reduced and allow them to save energy when foraging opportunities are scarce or energetically costly to pursue. Importantly, the chance of survival may be enhanced by torpor use, for example by decreasing foraging requirements and exposure to predators (Geiser and Brigham 2012). However, arousal from these states has been identified as energetically costly or physiologically challenging in many species (Currie et al. 2015; Landes et al. 2020). Therefore, for the use of daily torpor to be effective in managing energy requirements, animals need to balance the costs and benefits of torpor use against the benefits of foraging and the risks of predation and starvation (Jastroch et al. 2016).

Hibernation and daily torpor are widespread strategies in bats (Chiroptera). Due to their extreme energetic demands for maintaining flight, echolocation and thermoregulation (Lyman 1970; Winter and Von Helversen 1998; Currie et al. 2020), many bats are highly dependent upon temporal heterothermy to save energy during inclement conditions. Many bats are insectivorous, and thus depend upon food that varies seasonally with ambient temperature and weather (Stawski 2012a). The typical decreases in insect activity during winter have in previous studies been linked to a general reduction in the activity levels of bats during winter compared to summer (Richards 1989; Stawski and Geiser 2010b). As a result, seasonality is often used as a proxy for thermal conditions and for food availability and is, therefore, seen as a driver of torpor patterns (Wojciechowski et al. 2007; Geiser 2020). Food availability being a driver in itself is particularly evident when considering the contrary seasonal torpor patterns of the nectivorous subtropical blossom bat (*Syconycteris australis*), which uses more torpor during summer than winter as the flower nectar they feed on is more abundant during winter (Coburn and Geiser 1998).

For insectivorous bat species, multiple environmental conditions besides *T*_a_ have been found to affect nightly activity levels and foraging intensity. This includes effects of variation in precipitation, wind speed, humidity, barometric pressure and moonlight (Fenton et al. 1977; Paige 1995; Erickson and West 2002; Lang et al. 2006; Turbill 2008; Wolcott and Vulinec 2012; Appel et al. 2017), which have been linked to physiological or thermoregulatory costs, decreases in food abundance or increased predation risk, respectively. However, environmental conditions are not the only drivers of temporal patterns in activity and torpor. Behavioural decisions linked to trade-offs in energy allocation are strongly connected to the current state of an individual (McNamara and Houston 1996). State-dependent foraging behaviour and torpor use in bats have previously been linked to individual reproductive state (Hamilton and Barclay 1994; Mackie and Racey 2007), severity of infections (Reeder et al. 2012), and individual body condition (Park et al. 2000; Stawski and Geiser 2010a). Thus, in order to understand torpor decisions made at the individual level, both environmental conditions and individual state need to be considered.

With this study we aimed to explore what underlies the balance between nocturnal torpor use and foraging in insectivorous bats, using a large dataset collected on the eastern long-eared bat (*Nyctophilus bifax*). This is an insectivorous bat species endemic to the subtropical and tropical regions of Australia and has previously been found to employ torpor across seasons and climate zones (Stawski and Geiser 2010b; Stawski 2012b), indicating possible common individual torpor responses to changes in environmental conditions. Most studies investigating environmental effects on nightly bat activity tend to measure activity based upon capture rates, echolocation frequencies or emergence numbers from roosts. In this study, we instead explore the effect of nightly conditions on individual torpor use, which as a direct physiological response differs from indirect measures of activity levels (Wojciechowski et al. 2007; Salinas et al. 2014). Torpor use should thus tell us more about how these bats evaluate prospective foraging conditions and the relative costs and benefits to their energy budget and life history in order to employ torpor at specific times. We tested the hypothesis that torpor should be consistently used as a sensible response to inclement conditions that are likely to affect prey abundance and/or the bat’s energy expenditure in flight, as this would limit potential benefits of foraging. Additionally, we hypothesized that individual state and perceived predation risk (using moon illumination as a proxy here) would also impact nightly torpor use, again due to shifts in the balance between costs and benefits of active foraging versus rest using torpor.

## Materials and methods

### Data collection

Eastern long-eared bats were captured across seasons at one subtropical and one tropical field site in Australia between 2007 and 2009. At the southern subtropical location at Iluka Nature Reserve (New South Wales, 29°24′ S, 153°22′ E) bats were captured during the austral winter (July–August 2007, *N*_ind_ = 8), summer (February–March 2008, *N*_ind_ = 12) and spring (October–November 2008, *N*_ind_ = 6). At the northern tropical location in Djiru National Park (Queensland, 17°50′ S, 146°03′ E) bats were captured during two consecutive winters (June 2008, *N*_ind_ = 5; July–August 2009, *N*_ind_ = 6). The climate characteristics varied between the two sites, with the subtropical location (weather station number 058012) generally experiencing colder *T*_a_ (mean minimum and mean maximum *T*_a_ being, respectively, 9.7 and 19.1 °C in July, and 20.4 and 26.8 °C in February) than the tropical location (weather station number 032037; mean minimum and mean maximum *T*_a_ being respectively 15.2 and 23.9 °C in July, and 22.8 and 30.8 °C in February) when looking at climate statistics for the last 75–140 years (Australian Bureau of Meteorology). The subtropical location also received less than half of the mean annual rainfall (1462 mm) compared with the tropical location (3283 mm).

Permits for this study were approved by the University of New England Animal Ethics Committee (AEC08/046 and AEC09/058), New South Wales National Parks and Wildlife Service (no. S12448), and Queensland Parks and Wildlife Service (WITK04955708). Bats were captured using mist-nets placed within openings in the rainforest or across pathways. After capture, we trimmed a small patch of fur from the mid-dorsal region and attached a temperature-sensitive transmitter (~ 0.5 g, LB-2NT, Holohil Systems Inc., Carp, Ontario, Canada) with a skin adhesive (SkinBond, Smith and Nephew United, Mount Waverley, NSW, Australia). The bats were released at the capture site and tracked to their roost where we placed an antenna and a remote logger (Körtner and Geiser 1998), recording pulse intervals from the transmitters every 10 min. We calibrated transmitters between 5 and 40 °C in a water bath prior to attaching them to the bats, and the logged pulse intervals could afterwards be converted to skin temperatures (*T*_skin_).

### Data variables

**Fig. 1:**
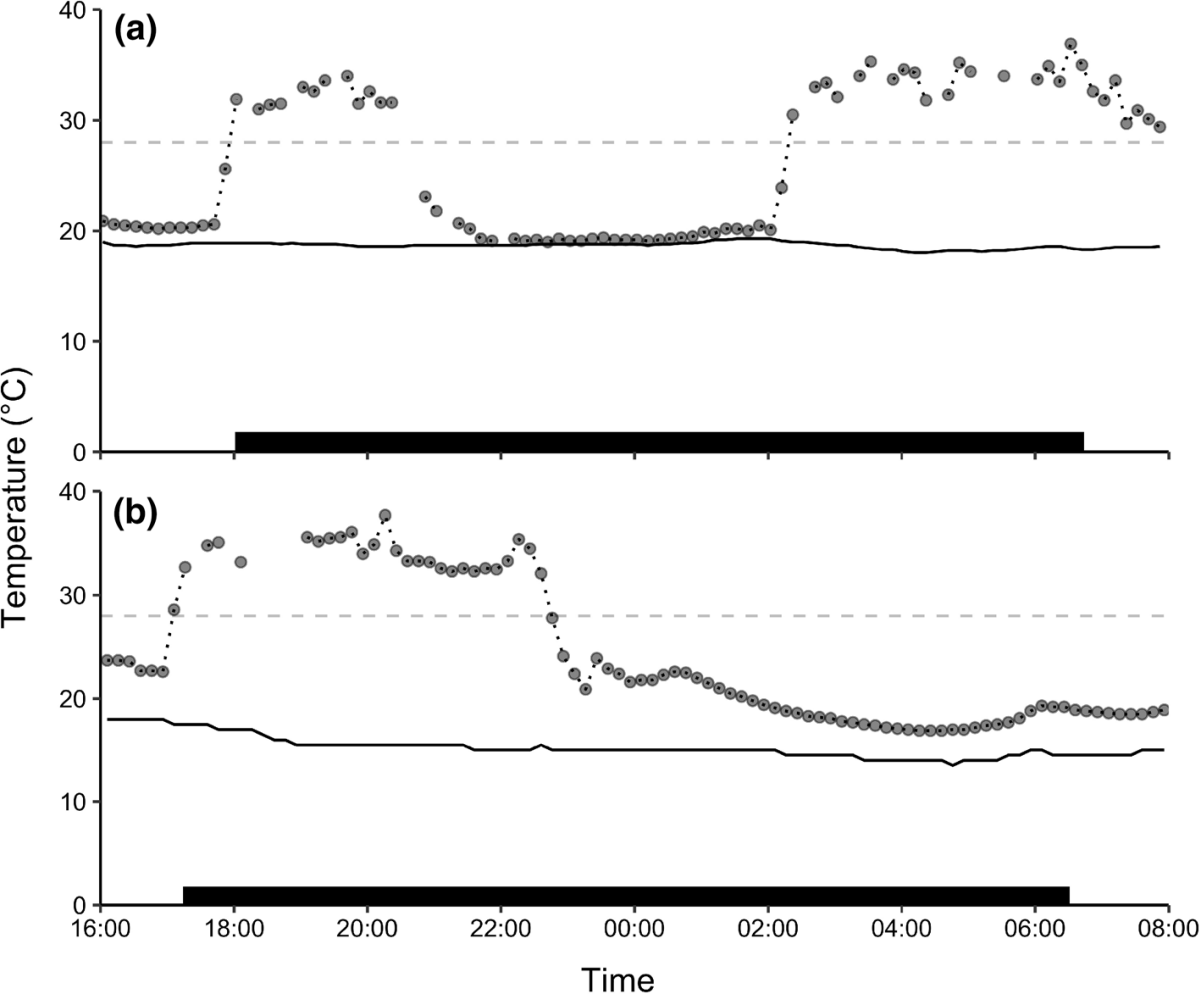
Skin temperature (*T*_skin_) patterns of two female eastern long-eared bats (dotted lines) over one measured winter night to illustrate torpor bouts expressed **a** at the tropical location and **b** at the subtropical location. Solid lines show the measured ambient temperature (*T*_a_) outside the roosts, and the horizontal dashed lines indicate the torpor cut-off value used here of 28 °C. The black bars at the bottom represent the night-time period between sunset and sunrise

From the *T*_skin_ data we estimated nightly torpor use. Previous studies have suggested that *T*_b_ < 30.0–31.0 °C should be defined as torpor events (Barclay et al. 2001). With *T*_b_ − *T*_skin_ typically being < 2.0 °C for small mammals, we defined torpor bouts as a period of more than 30 min with *T*_skin_ below 28.0 °C. We acknowledge the issues with using a single cut-off value to define torpor bouts (see Boyles et al. 2011). However, other methods also introduce uncertainty, and no consensus has been reached for deciding on the best method to determine torpor from *T*_skin_ measurements alone. Importantly, the bats in our study employed torpor bouts that decreased *T*_skin_ well below 28 °C in most cases, and although we cannot guarantee that there were no overlooked shallower torpor bouts, we believe this to be less likely during night-time than day-time due to generally lower *T*_a_ values (the nightly *T*_a_ ranges in our dataset were 4.0–22.6 °C in winter, 10.0–22.5 °C in spring, and 17.0–25.5 °C in summer). See Fig. 1 for visual examples of torpor bouts expressed at the tropical (Fig. 1a) and subtropical location (Fig. 1b). Nightly torpor use was estimated as the total duration in minutes spent torpid between sunset and sunrise. We obtained sunset and sunrise data from the geodetic calculator on the Geoscience Australia webpage. 270 bat nights were recorded across the 37 individuals; 151 nights for females (*N* = 20) and 119 nights for males (*N* = 17). Number of nights recorded per individual ranged from a single night (3 females and 2 males) up to 19 nights for females and 26 nights for males, with the median being 7 for the females and 4 for the males.

The Australian Bureau of Meteorology provided us with weather variables, including hourly precipitation, relative humidity, windspeed, and barometric pressure (BP). Additionally, we recorded environmental temperature (°C) at 10-min intervals using temperature-sensitive data loggers (0.5 °C, DS 1921G Thermochron iButtons, Maxim Integrated Products, Inc., Sunnyvale, CA, USA) placed outside of bat roosts at the data-collection sites. One last predictor, the percentage of moon disc illuminated, we obtained from the lunar calendar through the Calendar Australia webpage. Unfortunately, this variable does not account for potential additional variability in illumination caused by cloud cover, due to lack of data on this combination of factors. The different environmental variables were all considered relevant to include in our analyses as they have been found to impact foraging behaviour or physiological costs in small bats (see “Discussion” for literature citations). Each variable except moon illumination was converted into six versions: mean nightly condition, the standard deviation of the mean nightly condition, the maximum and minimum hourly value during the night, the range between the minimum and maximum value, and accumulated values throughout the night. Additionally, from the mean BP data variable, we created a ΔBP variable which captured the change in mean barometric pressure from the previous night.

### Statistical analyses

We performed analyses in the software R (version 3.5.2). All numerical variables were scaled and centred through the *scale* function. For data reduction purposes and to avoid model over-fitting, we performed principal component analyses (PCA) followed by a varimax rotation with the *principal* function from the “psych” package (Revelle 2017). However, none of the PCA models revealed any clear structure in the covariances that could be used to extract composite weather measures. We, therefore, continued with the analyses using only the nightly mean and range weather variables, keeping in mind the levels of covariance already identified (see Supplementary Materials 1 for covariance matrices).

We constructed linear mixed-effect models using the *lmer* function from the “lmerTest” package (Kuznetsova et al. 2017), with individual ID and date ID as random effects. Proportions of variance explained by the random effects were calculated using the *get_variance* functions from the “insight” package (Lüdecke et al. 2019). The effects of season and location on torpor use were tested separately and could not be included together in further models due to imbalance in the dataset (only winters were measured at the tropical location). The effect of night length was tested for but was excluded from further models as the limited variation in this variable had no apparent effect on nightly torpor use. We first constructed preliminary models including the various environmental variables by separately testing the mean and the range version of each variable to examine which was a better fit for further model selection. During this stage we also tested for non-linear quadratic effects, but there were none. We thus identified that the mean and linear versions of each of the numerical environmental variables that best explained variation in torpor use and proceeded to construct a global mixed-effect model. The nightly mean for the numerical variables ranged from 6.0 to 23.4 °C for *T*_a_ (scaled range − 2.06 to 1.85); 0.0 to 1.5 mm for precipitation (− 0.32 to 5.18); 3.9 to 32.2 m/s for windspeed (− 1.75 to 3.25); 998 to 1022 hPa for BP (− 2.92 to 2.04); − 10.0 to 6.7 hPa for ΔBP (− 3.15 to 2.18); 38.5 to 96.1% for humidity (− 2.67 to 1.38); 0 to 100% for moon size (− 2.17 to 1.36).

#### The original global model

The original global model on the scaled raw data variables included all two-way interactions between the different fixed effects: *T*_a_, sex, precipitation, humidity, windspeed, BP, ΔBP, and moon size. In order to investigate collinearity-issues in the models, we noted the variance inflation factor (vif) using the *vif* function from the “car” package (Fox and Weisberg 2018). Commonly, vif-values should be < 5 to avoid the need for correcting measures and < 10 to avoid removal of one of the correlated explanatory variables, although these limits have been disputed (O’Brien 2007). As the most complex models had strong collinearity issues, we performed model reduction based not only on *P* values and AIC-ranking (Forstmeier and Schielzeth 2011), but also on vif-values in the early stages of the model selection. The maximum vif-value for each of the ten highest ranked models are listed in the model selection tables (Supplementary Materials 2). A model was considered a better fit when the ΔAIC was reduced with > 2 (Burnham and Anderson 2002). In cases where two models had ΔAIC < 2, the model with the least degrees of freedom was considered the better fit, based on the concept of parsimony.

#### Within- versus among-subject effect models

As the individuals in this study were measured across two study sites and during different seasons, different individuals will have faced different average environmental conditions during data collection. However, we were primarily interested in any common reversibly plastic responses to environmental cues (i.e. within-subjects effects), as opposed to these among-subjects effects driven mainly by the mean differences in environmental conditions experienced by the different individuals. We, therefore, applied the methods described in van de Pol and Wright (2009) to our data in order to decompose within- versus among-subject effects in mixed models. Unfortunately, convergence issues (largely due to the among-subjects variation) prevented us from applying all aspects of the method to the original global best model, due to its complexity and various interaction terms (see “Results”). We, therefore, performed separate model selection (as above) for just the within-subjects effects in order to see if it included the same predictors as the original combined-effect best model. Similar model selection for just the among-subjects effects again resulted in serious model convergence issues that could not be resolved, probably due to the unbalanced data set in this case. We therefore performed the decomposition of within- versus among-subjects effects, as recommended by van de Pol and Wright (2009), on 7 separate and simpler models, each including temperature (as expected, *T*_a_ was the main predictor) and one other environmental variable (see Supplementary Materials 3).

#### State-dependence

Forearm length and body mass were each added as interaction effects with every predictor in the best original model and in the best within-subjects effect model. We did not create or test specific body condition index metrics, as this method has been the subject of widespread concern regarding its statistical and biological validity (García-Berthou 2001; Wilder et al. 2016). Instead, the effect of body condition was assessed via the effect of body mass in the models that also controlled for the effect of skeletal body size in the form of forearm length. Forearm length and body mass were not significantly correlated (see Supplementary Materials 5) and could safely be included together in all models. However, correlation issues emerged when including forearm and body mass together with sex in the models, as females in this dataset have significantly larger forearms (*P* < 0.01) and are significantly heavier (*P* < 0.001) than males. Thus, in order to test for the state-dependent effects of body size or body condition (i.e. body mass controlling for body size) we excluded sex from the models before proceeding to the model selection process. The body mass of the three pregnant females was not included in these analyses, as in these cases it represented more than energetic state in the form of fat reserves.

We also tested for potential effects of weather conditions from the previous night (*t* − 1) as this could affect the individuals’ current state at time *t*. We performed additional model selection using the best within-subjects variance model, where we added the environmental conditions at time *t* − 1 (*T*_a_, precipitation, humidity, windspeed, BP, ΔBP, and moon size) using the same model structure as with the environmental variables at time *t*, with all effects being simple additive ones, except for the precipitation–sex interaction. However, the presence of temporal autocorrelations between successive values of the different weather variables could result in apparent temporal autocorrelations in individual behaviour, leading to non-random residuals around individual temporal trendlines (Mitchell et al. 2020). Indeed, moon size, barometric pressure and humidity showed heavy temporal auto-correlations between the *t* and the *t* − 1 variables (see Supplementary Materials 5), restricting us to only applying one of each variable at time *t*. Additionally, we included torpor use at time *t* − 1 to investigate if there was any residual variation in individual torpor linked to the previous night use that was not explained by the environmental variables from a current or previous night.

## Results

### General results

Nightly torpor use in these eastern long-eared bats varied greatly and ranged from no torpor use to spending the full night torpid (0 to 818 min). The mean nightly individual torpor use was 294.9 ± 259.8 min (*N*_nights_ = 270, *N*_ind_ = 37).

**Fig. 2:**
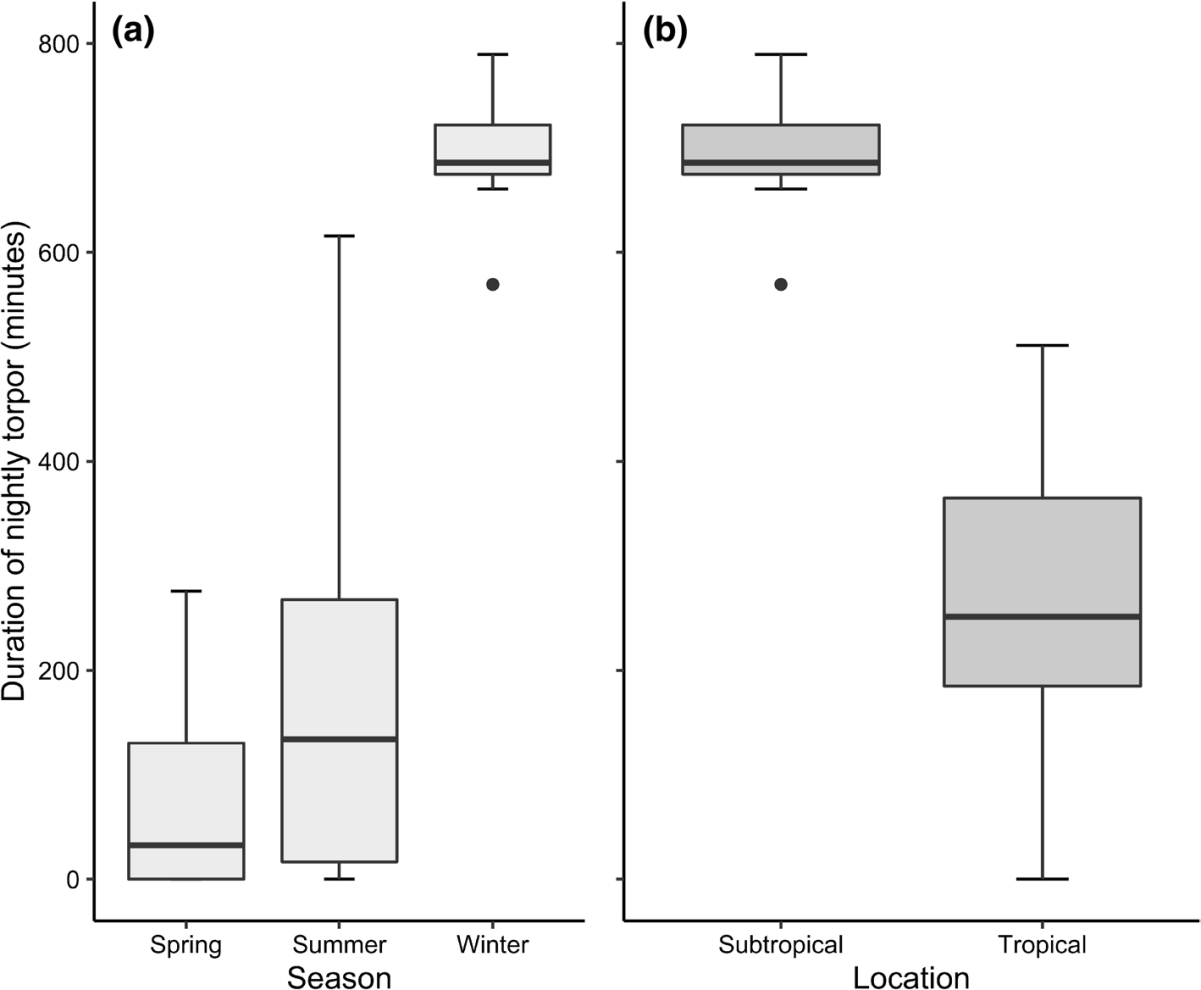
Individual nightly torpor use measured in eastern long-eared bats across seasons and locations. **a** Analyses of seasonal differences from the subtropical location revealed that the mean duration of torpor during the night was non-significantly different between spring and summer, but with significantly higher torpor use during winter. **b** The two locations measured during the winter season differed significantly in nightly mean torpor use with more nightly torpor expressed in the subtropical location compared to the tropical. Thick bars indicate median values, shaded boxes interquartile ranges, and whiskers the largest or smallest values within 1.5 times the interquartile ranges, with dots showing outlying values

Seasonal differences could only be tested with data from the subtropical location (*N*_nights_ = 197, *N*_ind_ = 26) and revealed a high seasonal variation in nightly torpor use (Fig. 2a). Mean individual nightly torpor use during spring and summer was not significantly different (spring 82.2 min, SE ± 58.1; summer 166.9 min ± 71.1; *P* = 0.25) but was significantly lower than the torpor use seen during the winter (692.5 min ± 76.8; *P* < 0.001). The difference in torpor use between the two locations could only be analysed using data from the winter (*N*_nights_ = 210, *N*_ind_ = 19) and showed a significantly (*P* < 0.001) higher individual mean nightly torpor use at the subtropical (692.5 min ± 42.9) compared to the tropical location (261.6 min ± 56.3; Fig. 2b).

### The original model

**Fig. 3:**
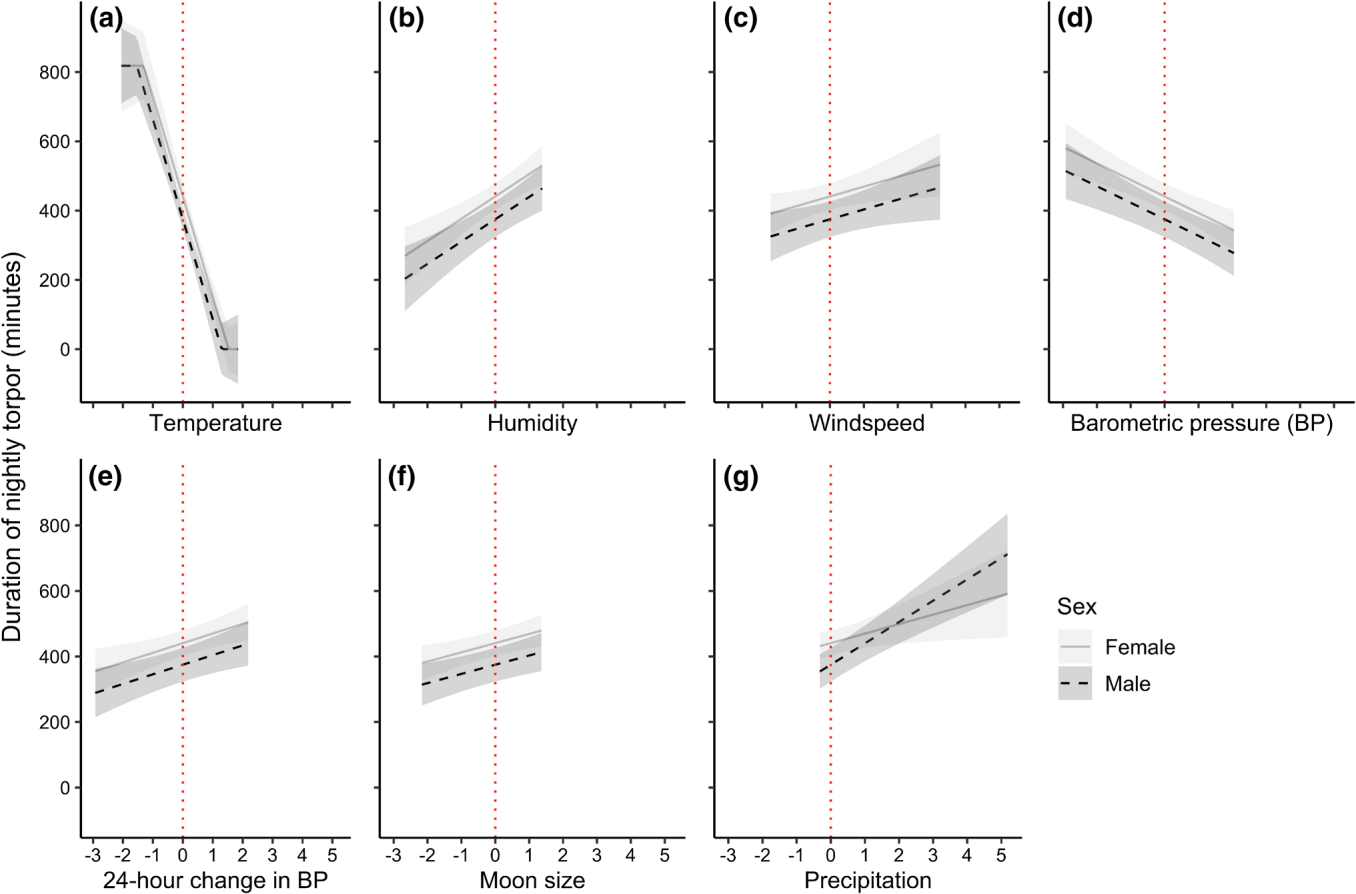
The main explanatory variable effects from the best model using the original variables (see Table 1) of **a** temperature, **b** humidity, **c** windspeed, **d** barometric pressure, **e** change in barometric pressure, **f** moon size, and **g** precipitation on the nightly torpor use in eastern long-eared bats, with the red dotted line indicating zero-centred values for each of these scaled predictors. Effects are shown for both males (black dashed best-fit lines and dark 95% CI shading) and females (grey solid best fit lines and light grey 95% CI shading). Only the precipitation-effect includes an interaction with sex, where the effect is stronger in males than in females

The overall model using the original variables that best explained the variation in nightly torpor use included all the explanatory variables plus several interactions (see Table 1, and Table S2.2 in Supplementary Materials 2 for the 10 highest ranked models). The random effect here of individual ID explained 5% of the total variation in torpor use, and day ID explained 3%, suggesting that the fixed effects included in this model explain the majority of the variation. Environmental variables that had a negative effect on nightly torpor duration included temperature and barometric pressure. Positive effects included precipitation, wind speed, ΔBP, humidity, and moon size. Additionally, females spent approximately an hour longer in torpor during the night than males. The interactions included a sex–precipitation effect where the positive precipitation effect on males was significantly stronger than on females. Additional interactions from the best model included a *T*_a_: ΔBP effect, a humidity:wind speed effect, a humidity:BP effect, and interactions between moon size and precipitation, BP, ΔBP, and humidity. See Table 1 for exact values and Fig. 3 for graphical presentations of these main results from the model. The interaction effects that included two weather condition predictors are presented graphically in Supplementary Materials 3 (Fig. S3.1). The scaling of the numerical values here allowed us to directly compare the estimated effects of each predictor on the nightly torpor duration in eastern long-eared bats. Nightly mean temperature (*T*_a_) was by far the strongest predictor, estimating an effect size more than four times larger than the second largest effect size.

**Table 1:** Estimates, standard error and *P* values of each explanatory variable included in the best model using the original variables, where the numerical predictors are scaled for direct comparison of their effect sizes on nightly torpor duration in eastern long-eared bats

Variable	Estimate	Std. error	*P* value
Random effects			
Day ID	0.03	0.0006	
Individual ID	0.05	0.0007	
Residual	0.08	0.001	
Fixed effects			
Intercept **♀**	440.8	19.4	< 0.001
Intercept **♂**	374.9	26.3	< 0.05
*T*_a_	− 288.3	15.0	< 0.001
Humidity	64.4	14.6	< 0.001
BP	− 47.8	10.6	< 0.001
ΔBP	29.4	9.6	< 0.01
Wind speed	28.3	13.0	< 0.05
Moon size	28.1	9.4	< 0.01
Precipitation **♀**	29.1	13.0	< 0.05
Precipitation **♂**	65.1	11.6	< 0.01
Moon size: humidity	44.1	10.1	< 0.001
Moon size: precipitation	− 39.9	16.7	< 0.05
Moon size: ΔBP	30.5	10.7	< 0.01
Humidity: BP	27.0	12.0	< 0.05
Humidity: wind speed	− 25.7	12.8	< 0.05
Moon size: BP	− 23.6	11.1	< 0.05
* T*_a_: ΔBP	− 22.1	7.9	< 0.01

The *P* values of the intercept and precipitation effect for males (♂) signifies whether the effects are different from the effect for females (♀). Day and individual ID were fitted as random effects and are given as the proportion of total variation explained

### Within- and among-subjects effect models

**Fig. 4:**
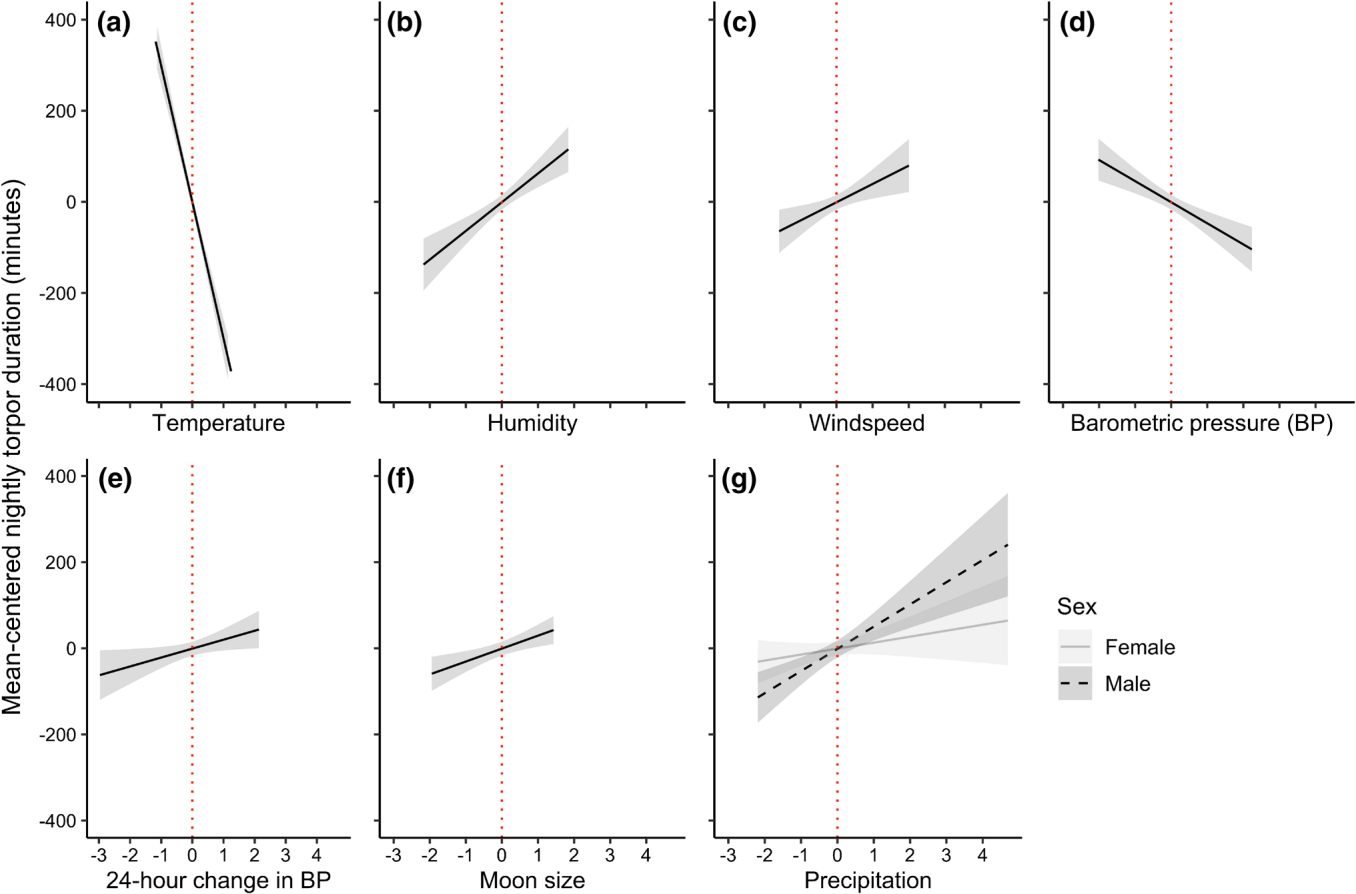
Within-subjects effects from the best model using individually mean-centred variables (see Table 2) of **a** temperature, **b** humidity, **c** windspeed, **d** barometric pressure, **e** change in barometric pressure, **f** moon size, and **g** precipitation on the nightly torpor use in eastern long-eared bats, with the red dotted line indicating zero-centred values for each of these scaled predictors. The precipitation-effect includes an interaction with sex, where the effect is non-significant in females (grey solid best fit lines and light grey 95% CI shading) but significant in males (black dashed best-fit lines and dark 95% CI shading)

The model selection of the within-subjects effects using individually mean-centred variables led to a simpler best model than the original model (above). All additive effects were still present in the final model, but of the interactions only the sex–precipitation interaction remained (Fig. 4). The sex effect was not significant by itself, but it had a significant interaction effect with precipitation, where males showed a stronger response to precipitation than females. Females were not significantly affected by precipitation. Similarly to the original model, the strongest predictor by far was the nightly *T*_a_, almost five times stronger than the second strongest predictor, which was humidity. The precipitation, barometric pressure and wind speed also showed strong effects on nightly torpor use (the precipitation effect was only significant on males), followed by moon size. Except for the non-significant effect of precipitation on females, ΔBP had the lowest effect size of the included parameters. See Table 2 for values and details.

**Table 2:** Estimates, standard error and *P* values of each variable included in the best within-subjects model based on individually mean-centred variables

Variable	Estimate	Std. error	*P* value
Random effects			
Day ID	0.10	0.002	
Residual	0.31	0.004	
Fixed effects			
Intercept **♀**	− 0.7	8.3	0.93
Intercept **♂**	− 1.3	10.1	0.96
* T*_a_	− 298.9	19.9	< 0.001
Humidity	63.0	12.9	< 0.001
BP	− 46.3	10.7	< 0.001
Wind speed	40.1	14.2	< 0.01
Moon size	30.0	9.7	< 0.01
ΔBP	20.7	9.5	< 0.05
Precipitation **♀**	13.9	11.1	0.21
Precipitation **♂**	51.5	12.9	< 0.01

As torpor use is mean-centred for each individual, the intercept is approximately 0 and Individual ID was excluded as a random effect

**Fig. 5:**
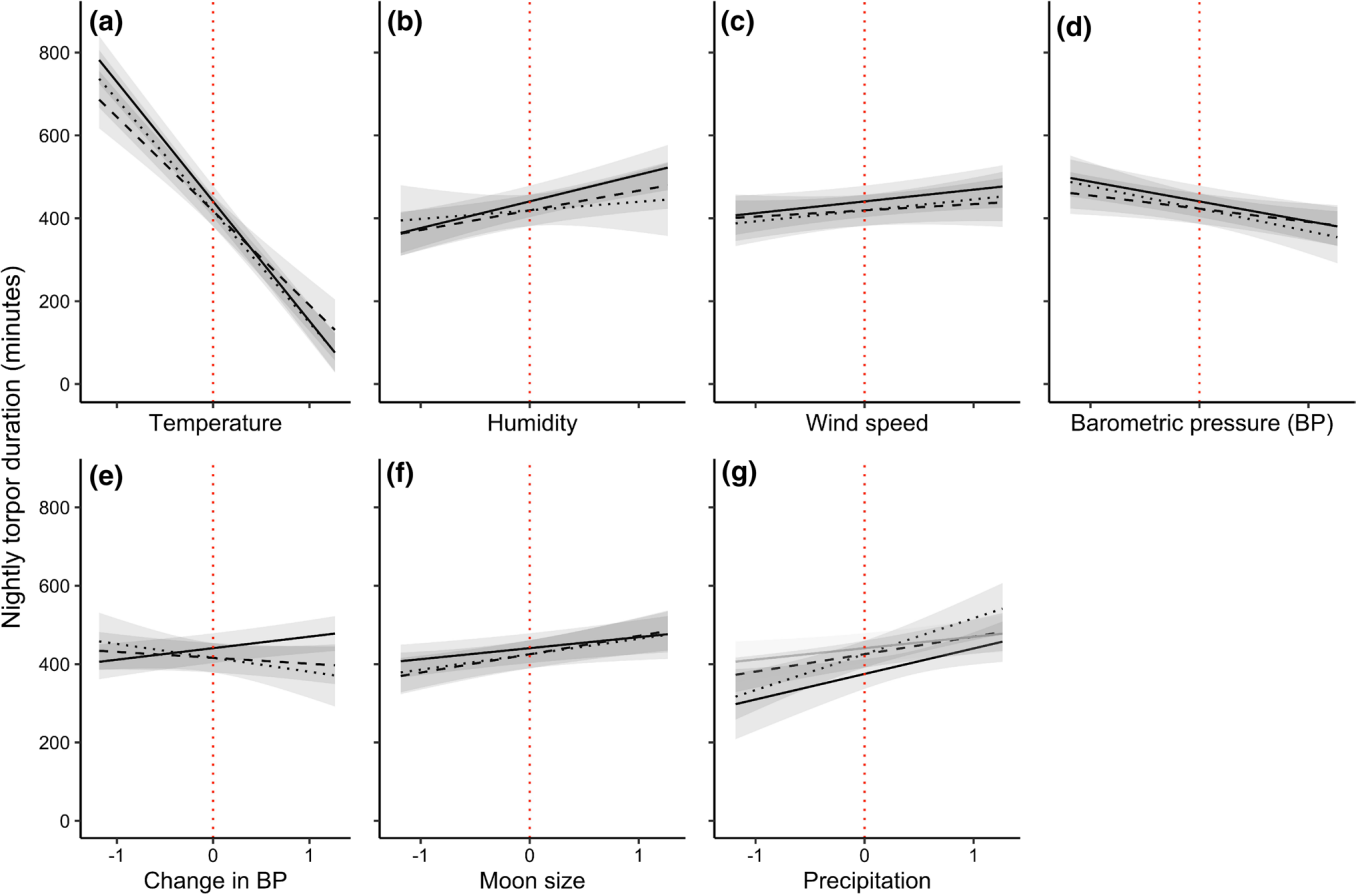
Comparing the original model effects (Table 1, solid lines and light grey CIs) with within-subjects effects (dashed lines and dark grey CIs) and among-subjects effects (dotted lines and medium shaded CIs) from simple models of **a** temperature, **b** humidity, **c** windspeed, **d** barometric pressure, **e** change in barometric pressure, **f** moon size, and **g** precipitation on the nightly torpor use in eastern long-eared bats. The effect of precipitation includes an interaction with sex, where the gray solid line represents females and the black solid line represents males. Red dotted lines indicate zero-centred values for each of the scaled predictors

In order to compare within- versus among-individual effects, we had to produce simpler models where we tested each variable separately (see "Methods"), and the results from each of these can be found in Supplementary Materials 4. Figure 5 illustrates the similarities between the overall additive effects from the original best model (Table 1) and the corresponding within- and among-subjects effects retrieved from these simple models. *T*_a_ remains the strongest predictor of nightly torpor use in eastern long-eared bats, indicating that all individuals responded in a similar manner to temperature changes, despite having been measured at different seasons and locations. Precipitation, another strong predictor, showed a significantly stronger among-subjects effect than within-subjects effect, although both effects were positive and significant in themselves. Different individuals, therefore, seem to respond by increasing nightly torpor use with increasing levels of precipitation, but part of the effect from the original model is caused by among-subject effects. However, as sex is not accounted for in the simple model it could explain part of why the among-subjects effect is greater than the within-subjects effect, as females were not significantly affected but males were. Another predictor revealing a difference in the effects was the ΔBP effect. The ΔBP effect from the original model was positive, which was also the case for the best within-subjects model, but the within- and between-subject ΔBP effects derived from the simpler model were slightly negative. This indicates that there are potential correlational issues with this variable that causes it to change its effect when it is modelled together with just *T*_a_ versus with additional predictors included. The remaining predictors showed similar original, within- and between-subjects effects, with only small differences (Fig. 5), revealing no apparent issues regarding correlation or over-fitting.

### State dependence

#### State dependence in the original model

The best original model including state variables contained the same interaction terms as the original model without state variables (Table 1), except for the moon:BP effect and the moon: ΔBP effect which were not present in the state-variable model. Additionally, the state-variable model included interactions between forearm length and precipitation and between body mass and wind speed, although forearm length and body mass did not show any significant effects on their own. This indicates that individuals with larger forearms (controlling for body mass) were more affected by precipitation than smaller individuals regarding the use of torpor at night, while individuals with a larger body mass (controlling for forearm length) were more affected by increasing nightly wind speeds. Effect sizes from the state-variable model are listed in Table 3, and graphical visualisations are shown in Figure S4.1.

**Table 3:** Estimates, standard error and *P* values of each variable included in the best model using the original explanatory variables, and including state-variables forearm length and body mass in place of sex

Variable	Estimate	Standard error	*P* value
Random effects			
Day ID	0.04	0.0007	
Individual ID	0.05	0.0007	
Residual	0.07	0.0009	
Fixed effects			
Intercept	403.3	18.4	< 0.001
*T*_a_	− 272.2	17.4	< 0.001
Humidity	44.8	16.7	< 0.01
Moon size	29.8	13.3	< 0.05
Precipitation	59.2	14.0	< 0.001
Wind speed	27.1	14.6	0.07
BP	− 37.4	12.7	< 0.01
ΔBP	24.2	12.4	0.06
Forearm length (FA)	22.9	15.4	0.15
Body mass (BM)	9.1	17.1	0.60
BM: wind	40.5	11.4	< 0.001
FA: precipitation	− 20.4	5.2	< 0.001
Humid: BP	40.5	16.1	< 0.05
Humid: wind	− 36.7	15.2	< 0.05
Humid: moon	32.6	13.4	< 0.05
Moon: precipitation	− 59.4	18.6	< 0.01
*T*_a_: ΔBP	− 24.8	10.2	< 0.01

#### State dependence in the within-subjects model

The best within-subjects model (Table 4), like the best original state-variable model (Table 3), showed no direct effects of body mass and forearm length, but included several interaction effects between the state variables and environmental effects as follows: individuals with shorter forearms or heavier body mass compared to conspecifics used more torpor during the night when precipitation levels increased, while individuals with a heavier body mass used more torpor with increasing wind speeds and ΔBP levels than light individuals (see Table 4 and Fig. S5.2). The best within-subjects model did not include any of the interaction terms between two environmental variables that were found in the best original state-variables model, but included two interaction terms that were not present in the original model. These were the body mass:precipitation effect and the body mass: ΔBP effect, revealing effects that may have been camouflaged in the original model by sex and overall environmental differences across seasons and locations. The two interaction terms present in both models (body mass:wind and forearm length:precipitation) showed similar effect sizes across the two models, although the forearm length:precipitation effect was slightly stronger in the within-subjects model, indicating that these effects are not caused by any among-subjects effects.

**Table 4:** Estimates, standard error and *P* values of each variable included in the best within-subject model using individual mean-centred variables, including state-variables forearm length and body mass in place of sex

Variable	Estimate	Standard error	*P* value
Random effects			
Day ID	0.12	0.002	
Residual	0.23	0.003	
Fixed effects			
Intercept	− 4.8	7.9	0.60
*T*_a_	− 312.9	21.3	< 0.001
Humidity	65.9	14.2	< 0.001
Moon size	54.6	11.7	< 0.001
Precipitation	39.5	11.1	< 0.001
Wind speed	38.5	15.8	< 0.05
BP	− 31.4	11.8	< 0.01
ΔBP	19.0	10.8	0.08
Forearm length (FA)	3.0	5.8	0.61
Body mass (BM)	0.6	8.9	0.95
BM: wind	37.1	17.7	< 0.05
BM: precipitation	31.3	14.2	< 0.05
FA: precipitation	− 28.9	5.9	< 0.001
BM: ΔBP	23.1	9.4	< 0.05

To further investigate possible state dependency, we also tested the effect of environmental conditions at time *t* − 1 to see if conditions on a previous night (and thus acquired individual differences in state) affected torpor use on the current night while accounting for current conditions. These analyses were complicated by a certain amount of temporal autocorrelations within some of the explanatory variables (Table S5.1 and Fig. S5.3). However, no *t* − 1 effects of these environmental variables could be identified, None of the models showed signs of state-dependent responses to the strongest predictor, nightly *T*_a_, which suggests that all individuals are equally affected by changes in temperature, regardless of their state. Nevertheless, scaled torpor use at time *t* − 1 did show a significant effect when included in the best within-subjects effect model (18.9 ± 6.8, *P* < 0.01, ΔAIC = − 5.8), where increasing levels of torpor on a previous night positively explained some of the residual variation in torpor use at time *t* (Fig. S5.4).

## Discussion

In this study we statistically disentangled the effects of multiple environmental factors on nightly torpor use on individual free-living bats across seasons and locations. The results reveal that Australian eastern long-eared bats use a variety of cues concerning the duration of their torpor use at night. As expected, mean nightly ambient temperature was by far the strongest predictor, more than four times larger than the second strongest predictor. The strong temperature effect across all individuals regardless of season, sex or state neatly explains the effects here of season and location. It also supports earlier findings of temperature being one of the main drivers of torpor behaviour in small endotherms, either due to the direct impact on thermoregulatory costs and/or by the indirect effect *T*_a_ has on insect prey availability (Twente and Twente 1965; Richards 1989; Ruf and Geiser 2015). However, the bats also responded by altering their nightly torpor use to environmental conditions like rain, wind, humidity, barometric pressure, and moon disk illumination, including some complex interactions between these effects. These results became clearer and more straightforward to understand when we considered only within-individual variation in these effects. We also found indications of state-dependent effects on torpor use, where body size moderated these individual responses to weather conditions, such as precipitation, windspeed, and change in barometric pressure. Contrary to what is currently known about torpor use during the resting phase in bats, we show here results indicating that torpor might be abandoned in face of too low energy reserves, as well as during inclement conditions like heavy rain or strong winds. Body size or state did not, however, affect the strongest responses involving ambient temperature, which remained the single main and unconfounded predictor of torpor use in these populations. Such a strong predictor, therefore, seems to affect individuals independent of their state, while other weather conditions may be evaluated by individuals based on their current state, such as fat reserves.

In contrast to the clear unconfounded effects of ambient temperature, the effects of precipitation were either sex, size or state dependent. Females were not significantly affected by variation in nightly rain conditions, while males increased their nightly torpor use with increasing precipitation levels. Replacing the sex–precipitation interaction term with body mass:precipitation and forearm length:precipitation in the best within-subject models improved the AIC value by 8.4. These interactions were also present at the within-subjects level, suggesting that these effects are in fact state- or size-dependent and not just driven by sex-specific differences. Similar individual state-dependent torpor responses have previously been found in mouse lemurs (Kobbe et al. 2011). Our findings indicate that smaller (male) bats with greater fat reserves might be able to respond to rainy conditions by saving stored energy and entering torpor, while larger (female) bats with lower levels of fat reserves cannot afford this and stay aroused to possibly take advantage of the opportunity to forage in between rain showers. It has been suggested that precipitation affects the activity levels in bats due to it interfering with the bats’ ability to echolocate and thus detect their prey (Griffin 1971) and by increasing their thermoregulatory demands as wet fur reduces its insulation value (Tuttle and Stevenson 1982). However, other studies have found that some rain conditions (mainly light or moderate precipitation) did not reduce activity levels in insectivorous bats (Kunz 1973; Hałat et al. 2018), perhaps because aerial insect abundance does not always decline during all types of rainfall in all habitat types. Even though precipitation has been investigated as a predictor of foraging activity in insectivorous bats, there is a lack of information about the effect of precipitation on bats’ use of nightly torpor. It is possible that the lack of response in torpor use to increasingly rainy conditions in females or bats with lower fat reserves does not necessarily mean that they spent more time foraging, because we did not analyse the time individuals spent away from the roost. However, as rainy conditions often vary in intensity throughout a day or night it is possible that these bats stayed aroused in order to benefit from potential rapid shifts in the weather. This was observed in a study by Fenton et al. (1977) where bat activity was supressed on rainy nights but only until the precipitation had tapered off, at which point bat activity resumed.

Wind speed may also rapidly shift in intensity throughout the night, which the mean wind condition variable will not have accounted for in our analyses. Wind speed has previously been found to negatively impact activity level in insectivorous bats (Avery 1985; Wolcott and Vulinec 2012) as well as increasing torpor expression in fishing bats (Salinas et al. 2014). The suggested mechanisms behind the effect of wind is that it functions as a source of increasing flight cost (Norberg 1990) and may also affect prey abundance by decreasing the number of flying insects (McGeachie 1989; Møller 2013). Increasing mean nightly wind speed lengthened the duration of nightly torpor in our eastern long-eared bats, but this effect was again dependent on individual body mass, both among and within individuals. As with precipitation, bats with lower body mass (while controlling for forearm length) did not respond to changes in mean nightly wind speed, while relatively heavier individuals with presumably greater fat reserves responded by using more torpor on more windy nights. It, therefore, appears that individuals with more fat reserves may have saved energy using extended bouts of torpor on nights with rain and strong winds, while individuals with less fat reserves are forced to forage or just stayed aroused, possibly to be ready to forage following shifts in the weather or even to forage regardless of conditions.

Barometric pressure is a variable that does not change as rapidly as precipitation or wind conditions, but indicates more general shifts in the weather. Somewhat surprisingly, higher nightly barometric pressures led to less torpor in our bats, but with few sex- or mass-dependent interactions with this effect. Conversely, a falling barometric pressure turned out to be apparently state-dependent, decreasing the torpor response in relatively heavy individuals, whilst relatively light individuals were unaffected. Consistent with our results, higher barometric pressure may be used by the bats as a proxy for good foraging conditions, leading to increased activity levels (Wolcott and Vulinec 2012; Bender and Hartman 2015), while falling barometric pressures have been shown to increase activity level in insectivorous bats, which has been linked to an increase in insect abundance (Paige 1995; Turbill 2008). However, our 24-h change in barometric pressure variable should perhaps be interpreted with caution, because the within-subjects effect was positive in the best within-subjects model when included alongside all the other effects (Fig. 4e), but negative in the simple models comparing amount- and within-subjects effect together in the same models that included temperature and only one other variable at the time (Fig. 5e). This indicates that, despite our efforts to control for covariance issues between our explanatory variables during our analyses, the effect shifts in this variable depending on whether it is modelled with other variables or only with *T*_a_, suggesting a complex series of interactions between environmental effects.

Increased relative humidity was found to lengthen nightly torpor duration in the eastern long-eared bats, independent of sex or individual state, and appeared as the second strongest predictor in the best within-subject effect model. Studies investigating nightly bat activity, however, report contradictory results, showing both greater bat activity with increasing relative humidity (Lacki 1984; Wolcott and Vulinec 2012) and lower bat activity with increasing relative humidity (O’Farrell and Bradley 1970). As we have analyzed data throughout seasons and locations, the overall and rather strong within-subjects humidity effect on nightly torpor use indicates that humidity conditions may be a more important driver of torpor use than previously reported, probably due to its negative effect on prey availability.

A topic that has caught the attention of many bat researchers is the effect of moonlight and/or lunar phase on bat activity. Here, we report a positive relationship between moon disk illumination and nightly torpor use in eastern long-eared bats. Many studies have previously investigated this effect and the results have been mixed, involving both negative effects of moonlight on bat activity and/or shifts to darker foraging habitats (Fenton et al. 1977; Lang et al. 2006; Appel et al. 2017), positive effects of moonlight on bat activity (Erickson and West 2002; Appel et al. 2017), or no effect at all (Karlsson et al. 2002; Holland et al. 2011). Some studies also point out shifts in insect abundance with lunar phases as a source of variation in the nightly activity patterns of insectivorous bats (Yela and Holyoak 1997; Lang et al. 2006). The variability and complexity of such moonlight effects on foraging success and/or predation risk suggests that this is likely to be highly species and habitat dependent. A review on anti-predator behaviour in bats by Lima and O’Keefe (2013), and the meta-analysis study on moonlight-avoidance by Saldaña-Vázquez and Munguía-Rosas (2013), both suggest that apparent ‘lunar phobia’ occurs mainly in tropical bat species. For temperate zone studies, there is little support for moonlight aversion in bats (Lima and O’Keefe 2013), and latitude was estimated to have a slight positive effect on lunar phobia across bat species (Saldaña-Vázquez and Munguía-Rosas 2013). The bats in our study, in both tropical and subtropical locations, showed a lunar phobic response by increasing their torpor use on nights with higher levels of moon disk illumination. This effect was surprisingly strong, comparable with other weather variables (excluding temperature and humidity), especially given that the variable did not account for potential variability in illumination due to cloud cover (see “Methods”). As other weather variables that may affect prey availability are accounted for in the analyses, our results show moon phases to be an important factor in individual bat nightly foraging decisions and energy budgeting across seasons and locations, potentially due to increased perceived predation risk under greater night-time illumination.

In this study, we have shown that across seasons and locations eastern long-eared bats in Australia employ torpor during the night as a consistent and predictable response to weather conditions and individual state. It appears that multiple environmental factors, as well as individual state (e.g. relative body mass, torpor the night before), are together taken into account in the use of night-time torpor versus active foraging or roosting. This species is endemic to the subtropical and tropical regions of Australia and faces a rapidly changing environment consistent with global trends. Many species have already shown responses to a changing climate by changing their distributional ranges, altering migration patterns or changing the timing of seasonal activities, potentially resulting in mismatching phenologies (IPCC 2014). However, temporal heterotherms may be buffered against certain costs of a changing climate by being more able to adjust their energy requirements through torpor and hibernation depending upon season and/or latitude. These are strategies that have been identified as key factors in reducing extinction risk in mammal species (Geiser and Turbill 2009; Liow et al. 2009). Hence, studies investigating the effect of climatic changes on long-term population trends in Europe found either weak or inconclusive effects on bat populations (Bowler et al. 2015; Martay et al. 2017). At the same time, bat populations are declining across a range of different species and environments, likely due to the cumulative effects of habitat loss, climate change, anthropogenic stressors and diseases (Rodhouse et al. 2012; Frick et al. 2019). Our results show how one bat species appears to strategically balance its energy budget by altering night-time torpor use when faced with varying weather conditions and individual state. In light of such phenotypic plasticity, it is currently unclear how much eastern long-eared bat populations and their distribution ranges will be affected by the predicted long-term increases in temperatures, droughts and shifts in atmospheric circulation on the east coast of Australia (Murphy and Timbal 2008). However, our results highlight the complexity and importance of weather conditions on insectivorous bat energy budgets, suggesting that the ongoing environmental change may have considerable impacts on the individual torpor and hibernation patterns across seasons and locations.

## Supplementary Information

Below is the link to the electronic supplementary material.

Supplementary file1 (DOCX 20344 KB)

## Data Availability

The datasets used and/or analysed during the current study are available from the corresponding author on reasonable request.
