# Supplementary material for: Nightly torpor use in response to weather conditions and individual state in an insectivorous bat
Source: Oecologia. 2021 Aug 28;197(1):129–42. doi: 10.1007/s00442-021-05022-6 (PMC8445878; doi:10.1007/s00442-021-05022-6)
Supplement: Supplementary file 1 — Supplementary file1 (DOCX 20344 KB) [file 442_2021_5022_MOESM1_ESM.docx]

Supplementary Materials 1: Correlation matrices

**Table S1.1**: Correlation matrix for the mean nightly variables. The number of asterisks indicate when p-values are < 0.5 (*), < 0.01 (**) and < 0.001 (***).

|  | Ta_Mean_ | Rain_Mean_ | Wind_Mean_ | Humid_Mean_ | BP_Mean_ | ΔBP |
| --- | --- | --- | --- | --- | --- | --- |
| Ta_Mean_ | 1 |  |  |  |  |  |
| Rain_Mean_ | 0.32 *** | 1 |  |  |  |  |
| Wind_Mean_ | 0.09 | 0.43 *** | 1 |  |  |  |
| Humid_Mean_ | 0.54 *** | 0.24 *** | -0.34 *** | 1 |  |  |
| BP_Mean_ | -0.18 ** | -0.29 *** | -0.34 *** | 0.21 *** | 1 |  |
| ΔBP | -0.14 * | -0.34 *** | -0.11 | -0.30 *** | 0.33 *** | 1 |
| Moon | -0.01 | 0.01 | 0.01 | 0.07 | -0.16 * | -0.12 * |

**Table S1.2**: Correlation matrix for the range nightly variables.

|  | Ta_Range_ | Rain_Range_ | Wind_Range_ | Humid_Range_ | BP_Range_ |
| --- | --- | --- | --- | --- | --- |
| Ta_Range_ | 1 |  |  |  |  |
| Rain_Range_ | -0.43 *** | 1 |  |  |  |
| Wind_Range_ | -0.12 * | 0.19 ** | 1 |  |  |
| Humid_Range_ | 0.35 *** | -0.15 * | 0.06 | 1 |  |
| BP_Range_ | -0.01 | 0.21 *** | 0.22 *** | 0.18 ** | 1 |

**Table S1.3**: Correlation matrix between the mean versus range nightly variables.

|  | Ta_Mean_ | Rain_Mean_ | Wind_Mean_ | Humid_Mean_ | BP_Mean_ |
| --- | --- | --- | --- | --- | --- |
| Ta_Range_ | -0.69 *** | -0.38 *** | 0.03 | -0.51 *** | -0.07 |
| Rain_Range_ | 0.36 *** | 0.89 *** | 0.25 *** | 0.27 *** | -0.17 ** |
| Wind_Range_ | 0.38 *** | 0.20 *** | 0.39 *** | -0.05 | -0.34 *** |
| Humid_Range_ | -0.19 ** | -0.22 *** | -0.09 | -0.08 | 0.01 |
| BP_Range_ | 0.25 *** | 0.27 *** | 0.32 *** | 0.03 | -0.53 *** |

Supplementary Materials 2: Model selection

Original Global Model:

The global original model was constructed with interaction effects between each parameter, which included sex, each of the mean environmental variables (T_a_, precipitation, wind speed, humidity and barometric pressure), ΔBP and moon disk illumination. Individual ID and date ID were included as random effects. The results from the global model are shown in Table S2.1, while Table S2.2 shows the 10 highest ranked models after performing the model selection.

**Table S2.1**: Estimates, standard error and p-values of each variable included in the global model using the original variables, where the numerical predictors are scaled for comparison of their effect on nightly torpor duration in eastern long-eared bats. p-values for males (♂) for each environmental variable indicate whether the effect was significantly different from females (♀). Day and individual ID were fitted as random effects and are given as the proportion of total variation explained.

| Variable | Estimate | Std. Error | p-value |
| --- | --- | --- | --- |
| *Random effects* | | | |
| Day ID | 0.02 | 0.0005 |  |
| Individual ID | 0.05 | 0.0008 |  |
| Residual | 0.07 | 0.001 |  |
| *Fixed effects* | | | |
| Intercept ♀ | 480.2 | 42.2 | < 0.001 |
| Intercept ♂ | 416.4 | 27.6 | < 0.05 |
| T_a_ ♀ | -291.4 | 33.2 | < 0.001 |
| T_a_ ♂ | -314.4 | 23.3 | 0.33 |
| Humidity ♀ | 49.6 | 24.3 | < 0.05 |
| Humidity ♂ | 68.5 | 18.4 | 0.31 |
| BP ♀ | -42.8 | 16.8 | < 0.05 |
| BP ♂ | -68.8 | 15.2 | 0.09 |
| ΔBP ♀ | 16.7 | 14.8 | 0.26 |
| ΔBP ♂ | 28.9 | 13.6 | 0.37 |
| Wind speed ♀ | 23.1 | 21.6 | 0.29 |
| Wind speed ♂ | 21.0 | 19.3 | 0.91 |
| Moon size ♀ | 17.3 | 16.2 | 0.29 |
| Moon size ♂ | 33.0 | 15.8 | 0.32 |
| Precipitation ♀ | 140.0 | 111.2 | 0.21 |
| Precipitation ♂ | 175.0 | 15.4 | < 0.05 |
| T_a_ : Humidity | 11.5 | 15.2 | 0.45 |
| T_a_ : BP | 17.6 | 10.9 | 0.11 |
| T_a_ : ΔBP | -30.3 | 11.6 | < 0.05 |
| T_a_ : Wind speed | -2.1 | 19.3 | 0.91 |
| T_a_ : Moon size | 15.8 | 13.9 | 0.26 |
| T_a_ : Precipitation | -68.2 | 89.4 | 0.45 |
| Humidity : BP | 19.6 | 14.4 | 0.18 |
| Humidity : ΔBP | 24.8 | 18.6 | 0.19 |
| Humidity : Wind speed | -21.2 | 14.7 | 0.15 |
| Humidity : Moon size | 39.8 | 17.2 | < 0.05 |
| Humidity : Precipitation | -75.5 | 56.5 | 0.18 |
| BP : ΔBP | -5.1 | 13.4 | 0.70 |
| BP : Wind speed | 12.6 | 17.3 | 0.47 |
| BP : Moon size | -19.8 | 11.6 | 0.09 |
| BP : Precipitation | -1.3 | 37.6 | 0.97 |
| ΔBP : Wind speed | 38.0 | 19.8 | 0.06 |
| ΔBP : Moon size | 26.2 | 10.8 | < 0.05 |
| ΔBP : Precipitation | -38.3 | 31.5 | 0.23 |
| Wind speed : Moon size | -6.9 | 14.0 | 0.62 |
| Wind speed : Precipitation | 11.8 | 41.2 | 0.77 |
| Moon size : Precipitation | -36.6 | 35.4 | 0.30 |

**Table S2.2**: The 10 highest ranked models derived from the model selection based on the original global model:

| Rank | Model | Max. VIF | df | AIC | ΔAIC |
| --- | --- | --- | --- | --- | --- |
| **1** | Torpor ~ Ta*ΔBP + Sex*Rain + Moon*(Rain + BP + Humidity + ΔBP) + Humidity*(Wind + BP) | 2.5 | 20 | 3278.14 | 0.0 |
| **2** | Torpor ~ Ta*(Moon + ΔBP) + Sex*Rain + Moon*(Rain + BP + Humidity + ΔBP) + Humidity*(Wind + BP) | 2.5 | 21 | 3278.98 | 0.8 |
| **3** | Torpor ~ Ta*(Moon + ΔBP) + Sex*(Rain + Moon) + Moon*(Rain + BP + Humidity + ΔBP) + Humidity*(Wind + BP) | 2.5 | 22 | 3279.41 | 1.3 |
| **4** | Torpor ~ Ta*(BP + Moon + ΔBP) + Sex*(Rain + Moon) + Moon*(Rain + BP + Humidity + ΔBP) + Humidity*(Wind + BP) | 2.9 | 23 | 3279.71 | 1.6 |
| **5** | Torpor ~ Ta*(BP + Moon + ΔBP) + Sex*(BP + Rain + Moon) + Moon*(Rain + BP + Humidity + ΔBP) + Humidity*(Wind + BP) | 2.9 | 24 | 3280.63 | 2.5 |
| **6** | Torpor ~ Ta*(Sex + BP + Moon + ΔBP) + Sex*(BP + Rain + Moon) + Moon*(Rain + BP + Humidity + ΔBP) + Humidity*(Wind + BP) | 2.9 | 25 | 3282.17 | 4.0 |
| **7** | Torpor ~ Ta*(Sex + BP + Moon + ΔBP) + Sex*(BP + Rain + Moon) + Moon*(Rain + BP + Humidity + ΔBP + Wind) + Humidity*(Wind + BP) | 3.5 | 26 | 3283.59 | 5.5 |
| **8** | Torpor ~ Ta*(Sex + BP + Moon + ΔBP) + Sex*(Humidity + BP + Rain + Moon) + Moon*(Rain + BP + Humidity + ΔBP + Wind) + Humidity*(Wind + BP) + Wind*ΔBP | 6.1 | 28 | 3285.02 | 6.9 |
| **9** | Torpor ~ Ta*(Sex + BP + Moon + ΔBP) + Sex*(Humidity + BP + Rain + Moon) + Moon*(Rain + BP + Humidity + ΔBP + Wind) + Humidity*(Wind + BP) | 3.6 | 27 | 3285.20 | 7.1 |
| **10** | Torpor ~ Ta*(Humidity + Sex + BP + Moon + ΔBP) + Sex*(Humidity + BP + Rain + Moon) + Moon*(Rain + BP + Humidity + ΔBP + Wind) + Humidity*(Wind + BP) + Wind*ΔBP | 6.1 | 29 | 3286.52 | 8.4 |

Within-Subjects Model:

The global within-subjects model using individually mean-centred variables was constructed in the same way as the global model using the original variables, with interaction terms between each fixed effect. Day ID was included as random effect. The results from the within-subjects model are shown in Table S2.3, while Table S2.4 shows the 10 highest ranked models after performing the model selection.

**Table S2.3**: Estimates, standard error and p-values of each variable included in the global within subjects model using individually mean-centred variables, where the numerical predictors are scaled for comparison of their effect on nightly torpor duration. p-values for males (♂) for each environmental variable indicate whether the effect was significantly different from females (♀).

| Variable | Estimate | Std. Error | p-value |
| --- | --- | --- | --- |
| *Random effects* | | | |
| Day ID | 0.07 | 0.002 |  |
| Residual | 0.30 | 0.004 |  |
| *Fixed effects* | | | |
| Intercept ♀ | 5.4 | 10.0 | 0.59 |
| Intercept ♂ | -2.8 | 10.1 | 0.42 |
| T_a_ ♀ | -283.0 | 24.4 | < 0.001 |
| T_a_ ♂ | -281.5 | 31.1 | 0.96 |
| Humidity ♀ | 72.6 | 15.7 | < 0.001 |
| Humidity ♂ | 70.4 | 19.5 | 0.91 |
| BP ♀ | -29.9 | 13.0 | < 0.05 |
| BP ♂ | -50.7 | 16.6 | 0.21 |
| ΔBP ♀ | 17.9 | 11.1 | 0.11 |
| ΔBP ♂ | 24.0 | 14.2 | 0.67 |
| Wind speed ♀ | 38.4 | 18.4 | < 0.05 |
| Wind speed ♂ | 28.9 | 22.2 | 0.67 |
| Moon size ♀ | 42.5 | 14.2 | < 0.01 |
| Moon size ♂ | 38.4 | 16.9 | 0.81 |
| Precipitation ♀ | 26.3 | 14.1 | 0.06 |
| Precipitation ♂ | 57.2 | 17.1 | 0.07 |
| T_a_ : Humidity | -8.1 | 27.9 | 0.77 |
| T_a_ : BP | 92.7 | 35.9 | < 0.05 |
| T_a_ : ΔBP | -66.7 | 41.8 | 0.11 |
| T_a_ : Wind speed | 48.8 | 43.4 | 0.26 |
| T_a_ : Moon size | 55.0 | 34.6 | 0.11 |
| T_a_ : Precipitation | -81.7 | 39.9 | < 0.05 |
| Humidity : BP | -21.4 | 14.9 | 0.16 |
| Humidity : ΔBP | 9.4 | 20.1 | 0.64 |
| Humidity : Wind speed | 25.9 | 29.3 | 0.38 |
| Humidity : Moon size | 24.6 | 20.4 | 0.23 |
| Humidity : Precipitation | -25.2 | 44.6 | 0.57 |
| BP : ΔBP | -6.5 | 13.2 | 0.62 |
| BP : Wind speed | 7.2 | 19.2 | 0.71 |
| BP : Moon size | -24.1 | 22.0 | 0.28 |
| BP : Precipitation | 9.7 | 29.9 | 0.75 |
| ΔBP : Wind speed | 5.7 | 23.4 | 0.81 |
| ΔBP : Moon size | 37.4 | 14.7 | < 0.05 |
| ΔBP : Precipitation | -34.4 | 20.1 | 0.09 |
| Wind speed : Moon size | 28.4 | 25.0 | 0.26 |
| Wind speed : Precipitation | -28.4 | 20.8 | 0.17 |
| Moon size : Precipitation | -2.8 | 20.8 | 0.89 |

**Table S2.4**: The 10 highest ranked models derived from the model selection based on the within-subjects global model. The best model was selected based on AIC ranking and the lowest degrees of freedom as there were several models with AIC-values < 2 points from the best model:

| Rank | Model | Max. VIF | df | AIC | ΔAIC |
| --- | --- | --- | --- | --- | --- |
| **1** | Torpor ~ Ta + Sex*Rain + Wind + BP + ΔBP + Humid + Moon | 1.8 | 12 | 3199.45 | 0.0 |
| **2** | Torpor ~ Ta*(Rain + Moon + BP) + Sex*Rain + Moon*(Wind + ΔBP + BP) + Humid | 2.1 | 19 | 3198.88 | -0.6 |
| **3** | Torpor ~ Ta*Rain + Sex*Rain + Wind + BP + ΔBP + Humid + Moon | 2 | 13 | 3198.99 | -0.5 |
| **4** | Torpor ~ Ta*(Rain + Moon + BP) + Sex*Rain + Moon*(Wind + ΔBP + BP) + Humid | 2.1 | 18 | 3199.08 | -0.4 |
| **5** | Torpor ~ Ta*(Rain + Moon) + Sex*Rain + Wind + BP + ΔBP + Humid + Moon | 2 | 14 | 3199.30 | -0.1 |
| **6** | Torpor ~ Ta*(Rain + Moon) + Sex*Rain + Moon*(Wind + ΔBP) + BP + Humid | 2 | 16 | 3199.35 | -0.1 |
| **7** | Torpor ~ Ta*(Rain + Moon) + Sex*Rain + Moon*Wind + BP + ΔBP + Humid | 2 | 15 | 3199.51 | 0.1 |
| **8** | Torpor ~ Ta*(Rain + Moon + BP + Wind) + Sex*(Rain + BP) + Moon*(Wind + ΔBP + BP + Humid) | 2.3 | 22 | 3199.71 | 0.3 |
| **9** | Torpor ~ Ta*(Rain + Moon + BP) + Sex*Rain + Moon*(Wind + ΔBP) + Humid | 2.1 | 17 | 3199.74 | 0.3 |
| **10** | Torpor ~ Ta*(Rain + Moon + BP + Wind) + Sex*Rain + Moon*(Wind + ΔBP + BP + Humid) | 2.3 | 21 | 3199.74 | 0.3 |

Original Model Including State Variables:

The global model using the original variables is presented here but with sex being replaced by forearm length and body mass (see Methods regarding covariance issues here), and including interaction terms between the two state-variables and each of the environmental variables, as well as three-way interaction terms between each of the state variables and the identified two-way interactions from the best original model (rank 1, Table S2.2). The results from the global model are shown in Table S2.5, while Table S2.6 shows the 10 highest ranked models after performing the model selection.

**Table S2.5**: Estimates, standard error and p-values of each variable included in the global model using the original explanatory variables, where the numerical predictors are scaled for comparison of their effect on nightly torpor duration.

| Variable | Estimate | Std. Error | p-value |
| --- | --- | --- | --- |
| *Random effects* | | | |
| Day ID | 0.03 | 0.0006 |  |
| Individual ID | 0.05 | 0.0008 |  |
| Residual | 0.05 | 0.0008 |  |
| *Fixed effects* | | | |
| Intercept | 415.3 | 19.1 | < 0.001 |
| T_a_ | -276.2 | 17.6 | < 0.001 |
| Humidity | 43.0 | 18.7 | < 0.05 |
| BP | -32.4 | 14.1 | < 0.05 |
| ΔBP | 19.0 | 13.7 | 0.17 |
| Wind speed | 21.8 | 15.2 | 0.15 |
| Moon size | 34.4 | 14.6 | < 0.05 |
| Precipitation | 65.0 | 13.9 | < 0.001 |
| Forearm length (FA) | 23.4 | 18.5 | 0.22 |
| Body mass (BM) | 19.1 | 22.5 | 0.40 |
| BM : Ta | 2.1 | 24.2 | 0.93 |
| BM : Humid | 3.4 | 21.3 | 0.87 |
| BM : BP | -4.9 | 14.5 | 0.73 |
| BM : ΔBP | 11.5 | 10.9 | 0.29 |
| BM : Wind | 20.2 | 16.3 | 0.22 |
| BM : Moon | 15.5 | 17.2 | 0.37 |
| BM : Precipitation | 43.8 | 23.8 | 0.07 |
| FA : Ta | 11.8 | 16.3 | 0.47 |
| FA : Humid | 12.3 | 13.8 | 0.37 |
| FA : BP | 8.2 | 12.6 | 0.52 |
| FA : ΔBP | -4.2 | 9.4 | 0.66 |
| FA : Wind | 4.0 | 12.0 | 0.74 |
| FA : Moon | -11.0 | 11.4 | 0.34 |
| FA : Precipitation | -38.9 | 11.5 | < 0.001 |
| T_a_ : ΔBP | -15.6 | 11.7 | 0.18 |
| Moon : Precipitation | -53.8 | 19.1 | < 0.01 |
| Moon : BP | -29.1 | 13.9 | < 0.05 |
| Moon : Humid | 50.5 | 15.3 | < 0.01 |
| Moon : ΔBP | 36.9 | 15.6 | < 0.05 |
| Humidity : Wind | -35.5 | 15.1 | < 0.05 |
| Humidity : BP | 32.2 | 18.1 | 0.08 |
| Ta : ΔBP : BM | -3.2 | 11.7 | 0.78 |
| Moon : Precipitation : BM | 51.1 | 41.0 | 0.22 |
| Moon : BP : BM | -49.7 | 17.6 | < 0.01 |
| Moon : Humid : BM | 28.4 | 23.7 | 0.23 |
| Moon : ΔBP : BM | 25.7 | 17.1 | 0.14 |
| Humidity : Wind : BM | -2.6 | 20.9 | 0.90 |
| Humidity : BP : BM | 0.1 | 15.2 | 0.99 |
| Ta : ΔBP : FA | -9.0 | 9.1 | 0.32 |
| Moon : Precipitation : FA | -0.4 | 15.7 | 0.98 |
| Moon : BP : FA | 18.9 | 15.5 | 0.23 |
| Moon : Humid : FA | -22.2 | 12.2 | 0.07 |
| Moon : ΔBP : FA | -31.0 | 13.7 | < 0.05 |
| Humidity : Wind : FA | 5.4 | 11.9 | 0.65 |
| Humidity : BP : FA | 10.1 | 14.4 | 0.48 |

**Table S2.6**: The 10 highest ranked models derived from the model selection based on the global model. The best model was selected based on AIC ranking and the lowest degrees of freedom as there were several models with AIC-values < 2 points from the best model (the second ranked model had an AIC-value > 2 points from the best model, but was ranked second based on the degrees of freedom and the significance-level of the removed parameters, which were non-significant):

| Rank | Model | Max. VIF | df | AIC | ΔAIC |
| --- | --- | --- | --- | --- | --- |
| **1** | Torpor ~ Ta*DBP + Moon*(Humid + Rain) + Humid*(BP + Wind) + FA*Rain + BM*Wind | 2.2 | 20 | 2610.22 | 0.0 |
| **2** | Torpor ~ Ta*DBP + Moon*(Humid + Rain) + Humid*(BP + Wind) + FA*(Rain + BP) + BM*(Wind + BP) | 2.2 | 22 | 2608.16 | -2.1 |
| **3** | Torpor ~ Ta*DBP + Moon*(Humid + Rain + ΔBP) + Humid*(BP + Wind) + FA*(Rain + BP) + BM*(Wind + BP) | 2.2 | 23 | 2609.09 | -1.1 |
| **4** | Torpor ~ Ta*DBP + Moon*(Humid + Rain + ΔBP) + Humid*(BP + Wind) + FA*(Rain + BP + ΔBP) + BM*(Wind + BP + ΔBP) | 2.2 | 25 | 2609.16 | -1.1 |
| **5** | Torpor ~ Ta*DBP + Moon*(Humid + Rain + ΔBP + BP) + Humid*(BP + Wind) + FA*(Rain + BP + ΔBP + Moon) + BM*(Wind + BP + ΔBP) | 2.5 | 27 | 2609.16 | -1.1 |
| **6** | Torpor ~ Ta*DBP + Moon*(Humid + Rain + ΔBP + BP) + Humid*(BP + Wind) + FA*(Rain + BP + ΔBP) + BM*(Wind + BP + ΔBP) | 2.5 | 26 | 2609.29 | -0.9 |
| **7** | Torpor ~ Ta*DBP + Moon*(Humid + Rain + ΔBP) + Humid*(BP + Wind) + FA*(Rain + BP) + BM*(Wind + BP + ΔBP) | 2.2 | 24 | 2609.35 | -0.9 |
| **8** | Torpor ~ Ta*DBP + Moon*(Humid + Rain) + Humid*(BP + Wind) + FA*(Rain + BP) + BM*(Wind) | 2.2 | 21 | 2529.79 | -0.5 |
| **9** | Torpor ~ Ta*DBP + Moon*(Humid + Rain + ΔBP + BP) + Humid*(BP + Wind) + FA*(Rain + BP + ΔBP + Moon) + BM*(Wind + BP + ΔBP + Moon) | 2.5 | 28 | 2610.04 | -0.2 |
| **10** | Torpor ~ Ta*DBP + Moon*(Humid + Rain + ΔBP + BP) + Humid*(BP + Wind) + FA*(Rain + BP + ΔBP + Moon) + BM*(Wind + BP + ΔBP + Moon + Rain) | 2.7 | 29 | 2610.96 | 0.7 |

Within-subject model with state variables:

The global within-subjects model using individually mean-centred explanatory variables is presented here with forearm length and body mass replacing sex (see Methods regarding covariance issues here), it included interaction terms between the two state-variables and each of the environmental variables. The results from the global model are shown in Table S2.7, while Table S2.8 shows the 10 highest ranked models after performing the model selection.

**Table S2.7**: Estimates, standard error and p-values of each variable included in the within-subjects global model using individually mean-centred explanatory variables, with body mass and fore-arm length in place of sex, where the numerical predictors are scaled for comparison of their effect on nightly torpor duration.

| Variable | Estimate | Std. Error | p-value |
| --- | --- | --- | --- |
| *Random effects* | | | |
| Day ID | 0.13 | 0.002 |  |
| Residual | 0.20 | 0.003 |  |
| *Fixed effects* | | | |
| Intercept | -5.2 | 7.9 | 0.51 |
| T_a_ | -314.4 | 22.0 | < 0.001 |
| Humidity | 65.6 | 15.7 | < 0.001 |
| BP | -34.7 | 13.1 | < 0.01 |
| ΔBP | 22.0 | 11.4 | 0.06 |
| Wind speed | 45.4 | 16.3 | < 0.01 |
| Moon size | 58.3 | 11.8 | < 0.001 |
| Precipitation | 38.1 | 11.3 | < 0.01 |
| Forearm length (FA) | 2.7 | 5.5 | 0.62 |
| Body mass (BM) | 0.6 | 8.5 | 0.95 |
| BM : Ta | -36.6 | 33.4 | 0.28 |
| BM : Humid | 1.1 | 19.5 | 0.96 |
| BM : BP | -12.4 | 14.7 | 0.40 |
| BM : ΔBP | 23.4 | 11.2 | < 0.05 |
| BM : Wind | 53.5 | 19.8 | < 0.01 |
| BM : Moon | 16.9 | 22.3 | 0.45 |
| BM : Precipitation | 24.5 | 18.2 | 0.18 |
| FA : Ta | 36.1 | 17.0 | < 0.05 |
| FA : Humid | -0.4 | 11.4 | 0.97 |
| FA : BP | 17.4 | 9.9 | 0.08 |
| FA : ΔBP | -6.9 | 8.8 | 0.43 |
| FA : Wind | -17.1 | 11.4 | 0.14 |
| FA : Moon | -17.1 | 8.9 | 0.06 |
| FA : Precipitation | -25.4 | 7.6 | < 0.01 |

**Table S2.8**: The 10 highest ranked models derived from the model selection based on the global within-subjects model. The best model was selected based on AIC ranking and the lowest degrees of freedom as there were several models with AIC-values < 2 points from the best model:

| Rank | Model | Max. VIF | df | AIC | ΔAIC |
| --- | --- | --- | --- | --- | --- |
| **1** | Torpor ~ BM*(Rain + Wind + DBP) + FA*Rain + Ta + BP + Humid + Moon | 3.2 | 16 | 2470.73 | 0.0 |
| **2** | Torpor ~ BM*(Rain + Wind + DBP) + FA*(Ta + Rain + Moon) + BP + Humid | 3.2 | 18 | 2469.02 | -1.7 |
| **3** | Torpor ~ BM*(Rain + Wind + DBP) + FA*(Rain + Moon) + Ta + BP + Humid | 3.2 | 17 | 2469.52 | -1.2 |
| **4** | Torpor ~ BM*(Rain + Wind + DBP) + FA*(Ta + Rain + Moon + BP) + Humid | 3.2 | 19 | 2469.67 | -1.1 |
| **5** | Torpor ~ BM*(Ta + Rain + Wind + DBP + BP) + FA*(Ta + Rain + Moon + Wind + BP) + Humid | 3.7 | 22 | 2469.90 | -0.8 |
| **6** | Torpor ~ BM*(Ta + Rain + Wind + DBP) + FA*(Ta + Rain + Moon + BP) + Humid | 3.4 | 20 | 2469.90 | -0.8 |
| **7** | Torpor ~ BM*(Ta + Rain + Wind + DBP + BP) + FA*(Ta + Rain + Moon + BP) + Humid | 3.6 | 21 | 2469.94 | -0.8 |
| **8** | Torpor ~ BM*(Ta + Rain + Moon + Wind + DBP + BP) + FA*(Ta + Rain + Moon + Wind + BP) + Humid | 4.0 | 23 | 2471.53 | 0.8 |
| **9** | Torpor ~ BM*(Ta + Rain + Moon + Wind + DBP + BP) + FA*(Ta + Rain + Moon + Wind + DBP + BP) + Humid | 4.0 | 24 | 2472.78 | 2.0 |
| **10** | Torpor ~ BM*(Rain + DBP) + FA*Rain + Ta + BP + Humid + Moon | 2.1 | 15 | 2472.90 | 2.2 |

Supplementary Material 3: Interaction effects from original model

The best global model using the original explanatory variables included 7 different interaction terms between the various environmental variables. These included interactions between moon size and 4 other variables (humidity, barometric pressure, ΔBP and precipitation), in addition to interactions between T_a_ and ΔBP, between humidity and wind, and between humidity and barometric pressure. See Table 1 in Results for effect-values, and Fig. S3.1 for the visualized interaction effects made by predicting the effect and confidence intervals across the observed predictor-range, using the delta-method. However, these interaction effects all disappeared from the best model when performing the model selection for the within-subject effects, indicating that they are likely to be caused by differences in environmental conditions measured for the different individuals (i.e. among-subject effects), and they should therefore be interpreted with caution.


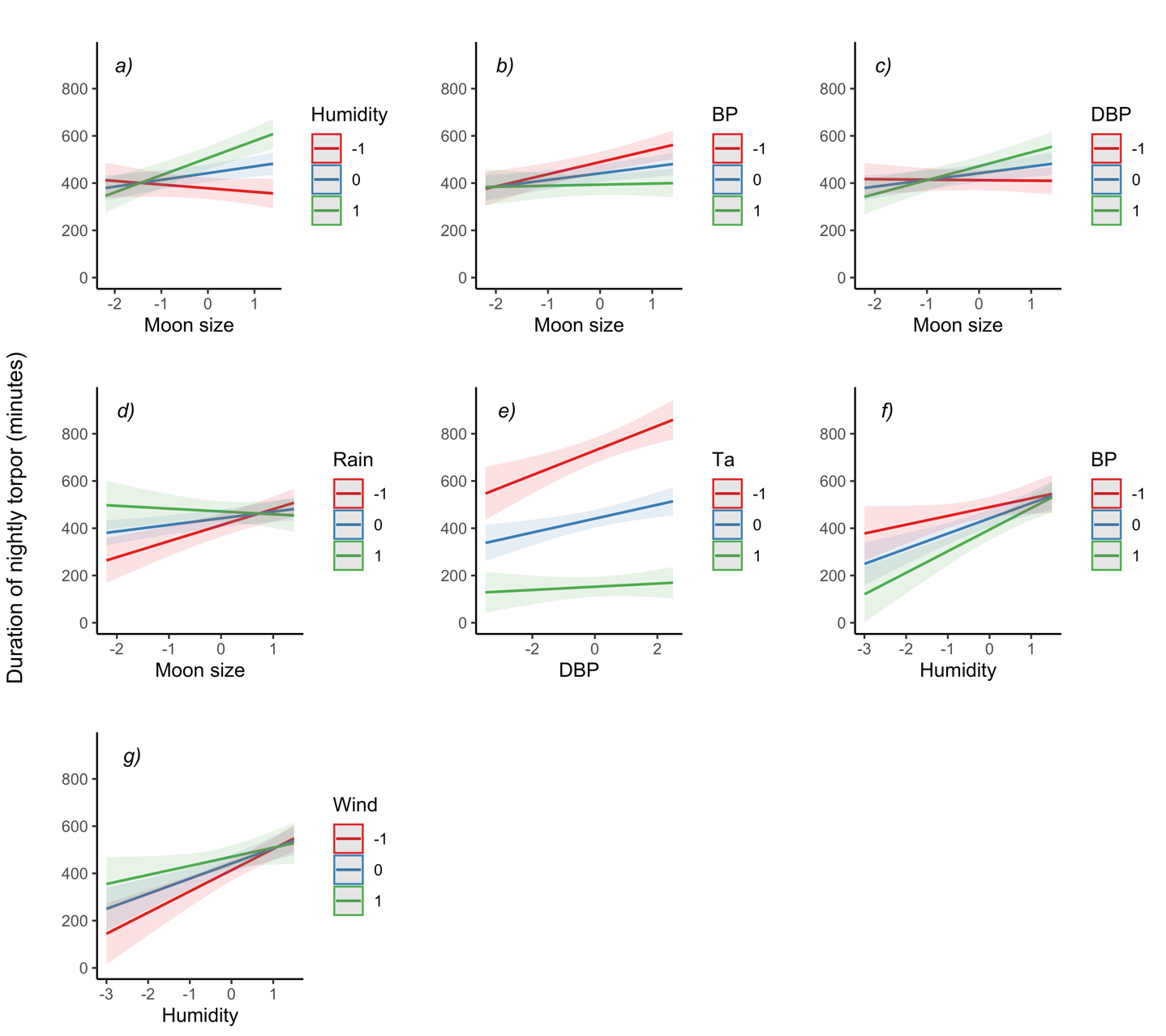


**Figure S3.1:** Interaction effects from the best global model for the different original explanatory variables. The colours correspond to the values 0 (blue), 1 (green), or -1 (red) for the scaled predictors explaining the effects. We chose to focus on the variables in the interaction which we believed to be the most novel or interesting when presenting each interaction-effect.

Supplementary Material 4: Within- vs. Among-Subject Effects

Due to convergence issues with the larger models when attempting to disentangle within- and among-subject effects (see Methods), we investigated these effects through the 7 simple models that are presented below. The models tested effects of one environmental variable at the time while accounting for the effects of T_a_, as this was the strongest predictor of nightly torpor use. To disentangle the within- vs. among-subject effects we followed the 3 steps described in van de Pol & Wright (2009).

Model 1: Torpor ~ T_a_

Model 2: Torpor ~ T_a_ + Precipitation

Model 3: Torpor ~ T_a_ + Wind

Model 4: Torpor ~ T_a_ + BP

Model 5: Torpor ~ T_a_ + ΔBP

Model 6: Torpor ~ T_a_ + Humidity

Model 7: Torpor ~ T_a_ + Moon size

Model results are shown in the Tables S4.1 to S4.7 with values for each of the three steps presented (original; within subjects (W); among subjects (A)). Black values indicate significant effects (p < 0.05), while red values are non-significant. The results show that the strongest predictor, T_a_, has a consistent effect in all the models (overall effect ranging from -253.0 to -271.3), which suggests that no interaction effects are causing major issues with this predictor. Further, the results show a non-significant difference between within- and among-subject effects for all the environmental effects except for precipitation, where the among-subject effect was stronger than the within-subject effect, although both showed significant effects in themselves. This indicates that part of the precipitation effect found in the original model is caused by among-subject effects.

**Table S4.1**: Model results from model 1 for the three models (step 1-3) tested in order to disentangle within- and among-subject effects. Standard deviation (random effects) and standard error (fixed effects) are shown in parentheses. Day and individual ID were fitted as random effects and are given as the proportion of total variation explained. Black values are significant (p < 0.05), while red values are non-significant.

Model 1: Torpor ~ T_a_

|  | Step 1 (original) | Step 2 (W. & A.) | Step 3 (W. vs. A) |
| --- | --- | --- | --- |
| *Random effects* | | | |
| Day ID | 0.09 (±0.001) | 0.09 (±0.001) | 0.09 (±0.001) |
| Individual ID | 0.08 (±0.0009) | 0.07 (±0.0009) | 0.07 (±0.0009) |
| Residual | 0.08 (±0.001) | 0.08 (±0.0009) | 0.08 (±0.0009) |
| *Fixed effects* | | | |
| Intercept | 411.1 (±18.7) | 417.7 (±19.0) | 417.7 (±19.0) |
| T_a_ | -253.0 (±16.0) |  |  |
| T_a_(W) |  | -226.9 (±25.4) | -226.9 (±25.4) |
| T_a_(A) |  | -269.3 (±19.8) |  |
| T_a_(A-W) |  |  | -42.4 (±32.0) |

**Table S4.2**: Model results from model 2 for the three models (step 1-3) tested in order to disentangle within- and among-subject effects.

Model 2: Torpor ~ T_a_ + Precipitation

|  | Step 1 (original) | Step 2 (W. & A.) | Step 3 (W. vs. A) |
| --- | --- | --- | --- |
| *Random effects* | | | |
| Day ID | 0.07 (±0.001) | 0.07 (±0.0009) | 0.07 (±0.0009) |
| Individual ID | 0.08 (±0.001) | 0.06 (±0.0008) | 0.06 (±0.0008) |
| Residual | 0.09 (±0.001) | 0.08 (±0.001) | 0.08 (±0.001) |
| *Fixed effects* | | | |
| Intercept | 416.6 (±18.0) | 425.5 (±17.5) | 425.5 (±17.5) |
| T_a_ | -261.6 (±5.2) |  |  |
| T_a_(W) |  | -235.3 (±23.1) | -235.3 (±23.1) |
| T_a_(A) |  | -294.3 (±19.5) |  |
| T_a_(A-W) |  |  | -59.0 (±29.8) |
| Rain | 50.7 (±12.9) |  |  |
| Rain (W) |  | 44.6 (±12.8) | 44.6 (±12.9) |
| Rain (A) |  | 91.7 (±21.4) |  |
| Rain (A-W) |  |  | 47.1 (±20.4) |

**Table S4.3**: Model results from model 3 for the three models (step 1-3) tested in order to disentangle within- and among-subject effects.

Model 3: Torpor ~ T_a_ + Wind

|  | Step 1 (original) | Step 2 (W. & A.) | Step 3 (W. vs. A) |
| --- | --- | --- | --- |
| *Random effects* | | | |
| Day ID | 0.09 (±0.001) | 0.08 (±0.0009) | 0.08 (±0.0009) |
| Individual ID | 0.08 (±0.0009) | 0.07 (±0.0009) | 0.07 (±0.0009) |
| Residual | 0.08 (±0.001) | 0.08 (±0.0009) | 0.08 (±0.0009) |
| *Fixed effects* | | | |
| Intercept | 413.1 (±18.8) | 419.2 (±19.1) | 419.2 (±19.1) |
| T_a_ | -256.0 (±16.0) |  |  |
| T_a_(W) |  | -231.4 (±25.4) | -231.4 (±25.5) |
| T_a_(A) |  | -271.7 (±20.1) |  |
| T_a_(A-W) |  |  | -40.3 (±32.2) |
| Wind | 22.4 (±13.9) |  |  |
| Wind (W) |  | 15.1 (±18.2) | 15.1 (±18.2) |
| Wind (A) |  | 26.5 (±18.0) |  |
| Wind (A-W) |  |  | 11.3 (±23.2) |

**Table S4.4**: Model results from model 4 for the three models (step 1-3) tested in order to disentangle within- and among-subject effects.

Model 4: Torpor ~ T_a_ + BP

|  | Step 1 (original) | Step 2 (W. & A.) | Step 3 (W. vs. A) |
| --- | --- | --- | --- |
| *Random effects* | | | |
| Day ID | 0.08 (±0.0009) | 0.07 (±0.0009) | 0.07 (±0.0009) |
| Individual ID | 0.07 (±0.0009) | 0.07 (±0.0008) | 0.07 (±0.0008) |
| Residual | 0.08 (±0.0009) | 0.07 (±0.0009) | 0.07 (±0.0009) |
| *Fixed effects* | | | |
| Intercept | 416.1 (±18.4) | 423.2 (±18.8) | 423.2 (±18.8) |
| T_a_ | -263.2 (±15.9) |  |  |
| T_a_(W) |  | -236.2 (±24.5) | -236.2 (±24.5) |
| T_a_(A) |  | -284.6 (±20.5) |  |
| T_a_(A-W) |  |  | -48.3 (±31.7) |
| BP | -38.3 (±12.4) |  |  |
| BP (W) |  | -32.0 (±14.0) | -32.0 (±14.0) |
| BP (A) |  | -54.4 (±21.5) |  |
| BP (A-W) |  |  | -22.4 (±24.4) |

**Table S4.5**: Model results from model 5 for the three models (step 1-3) tested in order to disentangle within- and among-subject effects.

Model 5: Torpor ~ T_a_ + ΔBP

|  | Step 1 (original) | Step 2 (W. & A.) | Step 3 (W. vs. A) |
| --- | --- | --- | --- |
| *Random effects* | | | |
| Day ID | 0.09 (±0.001) | 0.08 (±0.0009) | 0.08 (±0.0009) |
| Individual ID | 0.08 (±0.0009) | 0.07 (±0.0009) | 0.07 (±0.0009) |
| Residual | 0.08 (±0.001) | 0.07 (±0.0009) | 0.07 (±0.0009) |
| *Fixed effects* | | | |
| Intercept | 410.9 (±18.8) | 416.2 (±19.2) | 416.2 (±19.2) |
| T_a_ | -255.4 (±16.2) |  |  |
| T_a_(W) |  | -229.1 (±25.9) | -229.1 (±25.9) |
| T_a_(A) |  | -273.4 (±20.4) |  |
| T_a_(A-W) |  |  | -44.3 (±32.9) |
| ΔBP | -19.5 (±11.9) |  |  |
| ΔBP (W) |  | -15.1 (±12.4) | -15.1 (±12.4) |
| ΔBP (A) |  | -35.5(±27.6) |  |
| ΔBP (A-W) |  |  | -20.4 (±28.0) |

**Table S4.6**: Model results from model 6 for the three models (step 1-3) tested in order to disentangle within- and among-subject effects.

Model 6: Torpor ~ T_a_ + Humidity

|  | Step 1 (original) | Step 2 (W. & A.) | Step 3 (W. vs. A) |
| --- | --- | --- | --- |
| *Random effects* | | | |
| Day ID | 0.08 (±0.001) | 0.08 (±0.0009) | 0.08 (±0.0009) |
| Individual ID | 0.09 (±0.001) | 0.08 (±0.0009) | 0.08 (±0.0009) |
| Residual | 0.08 (±0.001) | 0.08 (±0.0009) | 0.08 (±0.0009) |
| *Fixed effects* | | | |
| Intercept | 413.1 (±19.1) | 419.2 (±19.4) | 419.2 (±19.4) |
| T_a_ | -271.3 (±17.3) |  |  |
| T_a_(W) |  | -254.9 (±26.0) | -254.9 (±26.0) |
| T_a_(A) |  | -275.5 (±24.0) |  |
| T_a_(A-W) |  |  | -20.6 (±35.2) |
| Humid | 44.4 (±15.0) |  |  |
| Humid (W) |  | 47.6 (±16.4) | 47.6 (±16.4) |
| Humid (A) |  | 20.7 (±32.4) |  |
| Humid (A-W) |  |  | -26.9 (±35.3) |

**Table S4.7**: Model results from model 7 for the three models (step 1-3) tested in order to disentangle within- and among-subject effects.

Model 7: Torpor ~ T_a_ + Moon

|  | Step 1 (original) | Step 2 (W. & A.) | Step 3 (W. vs. A) |
| --- | --- | --- | --- |
| *Random effects* | | | |
| Day ID | 0.08 (±0.0009) | 0.08 (±0.0009) | 0.08 (±0.0009) |
| Individual ID | 0.06 (±0.0009) | 0.06 (±0.0008) | 0.06 (±0.0008) |
| Residual | 0.08 (±0.0009) | 0.07 (±0.0009) | 0.07 (±0.0009) |
| *Fixed effects* | | | |
| Intercept | 423.5 (±17.9) | 425.3 (±18.3) | 425.3 (±18.3) |
| T_a_ | -258.5 (±15.2) |  |  |
| T_a_(W) |  | -247.8 (±25.2) | -247.8 (±25.2) |
| T_a_(A) |  | -266.6 (±19.1) |  |
| T_a_(A-W) |  |  | -18.8 (±31.2) |
| Moon | 45.9 (±11.5) |  |  |
| Moon (W) |  | 46.9 (±13.4) | 46.9 (±13.4) |
| Moon (A) |  | 39.4 (±18.1) |  |
| Moon (A-W) |  |  | -7.5 (±20.3) |

Supplementary Material 5: State Dependence

*Body Mass and Forearm Length*

As forearm length and body mass were not significantly correlated (0.31, p = 0.07) they could both be included in the models when performing the model selection. However, correlation issues emerged when including forearm and body mass together with sex in the models, as females in this species and sample have significantly larger forearms (1.34 ± 0.44, p < 0.01) and are significantly heavier (1.96 ± 0.34, p < 0.001) than males. In order to investigate state dependency on nightly torpor employed by eastern long-eared bats we replaced body mass and forearm length with sex and performed a model selection based on a global original model and a global within-subject model (see table S2.6 and S2.8 in Supplementary Materials 2 for the 10 highest ranked models from the two model selections).

Global Best Model

The best global model based on the original explanatory variables included all the predictors and interaction effects between body mass and wind speed, between forearm length and precipitation, between T_a_ and ΔBP, between humidity and barometric pressure, between humidity and wind speed, between moon size and precipitation and between moon size and humidity (see Table 3 in Results for effect values). The interaction effects are visualized in Fig. S5.1.


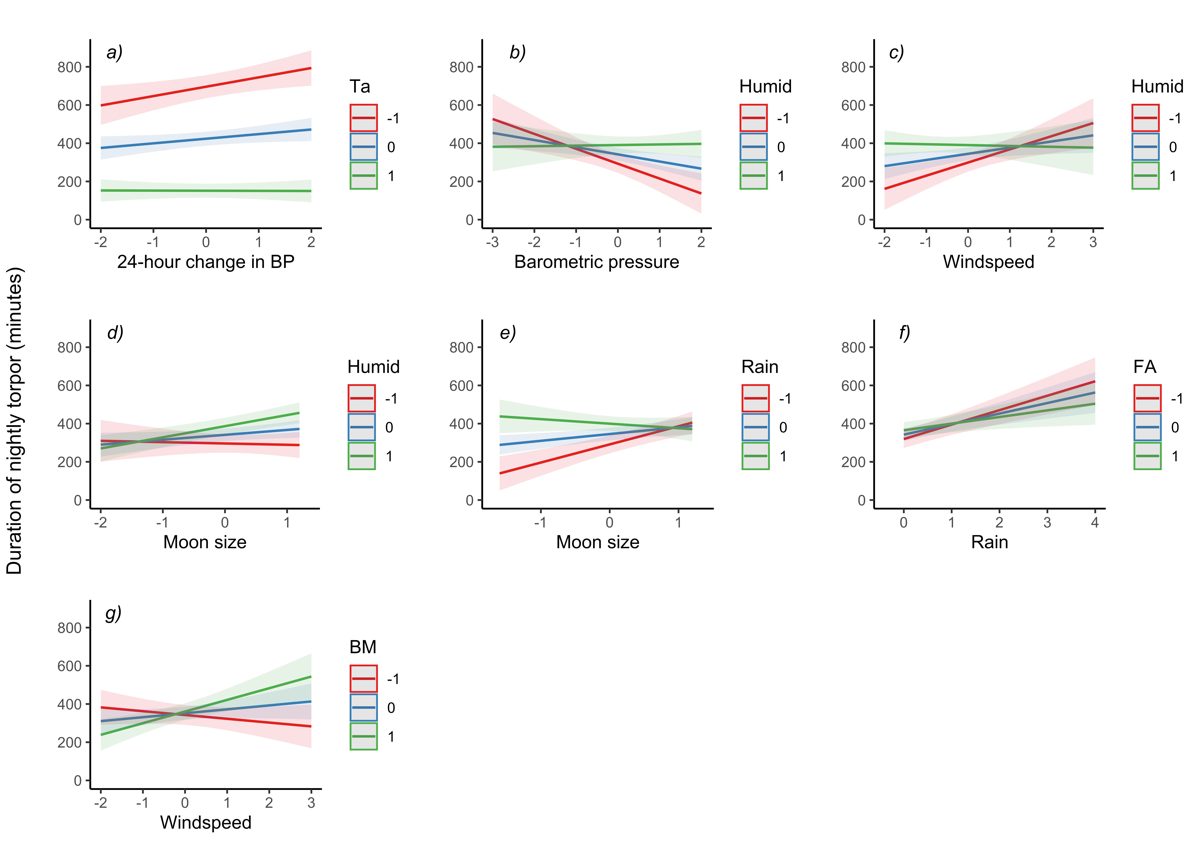


**Figure S5.1:** Interaction effects from the best global state model using the original explanatory variables including forearm length and body mass and their effects on weather condition impacts on the duration of nightly torpor, along with the interaction effects between environmental predictors. The colours correspond to the values 0 (blue), 1 (green), or -1 (red) for the scaled predictors explaining the effects.

Within-Subjects Model

The best within-subjects model using individually mean-centred explanatory variables included all the predictors and interaction effects between body mass and three of the environmental variables (wind speed, ΔBP, and precipitation) and between forearm length and precipitation (see Table 4 in Results for effect values). The interaction effects are visualized in Fig. S5.2.


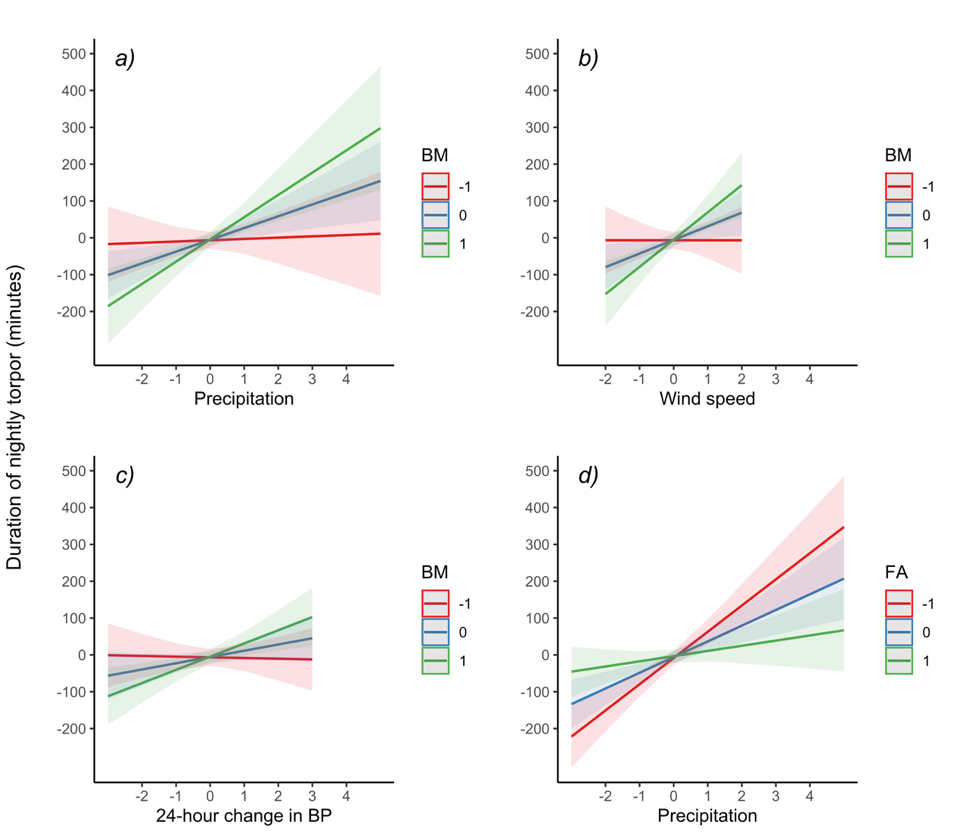


**Figure S5.2a-d:** Interaction effects from the best within-subject model including forearm length and body mass (in place of sex) and their effects on weather condition impacts on the duration of nightly torpor. The colours correspond to the values 0 (blue), 1 (green), or -1 (red) for the scaled predictors explaining the effects.

*Weather Conditions from the Night Before and Temporal Autocorrelation*

Moon disk illumination, barometric pressure and humidity showed strong temporal autocorrelations between their values at times t and t-1, which meant that only one of each version could be added at a time to the model. For humidity, the model including the values at t variable fitted the data better than the model including the values at t-1 variable (ΔAIC = 20.6). The models testing barometric pressure at time t and t-1 showed a slightly better fit for the model including the values at t variable, although the effects were similar in the two models (ΔAIC = 2.6). For the models including moon disk illumination at time t and time t-1, the model including moon light conditions from the previous night was a slightly better fit than the model with the values at t variable (ΔAIC = 1.8), but the effect sizes were almost identical.

In order to establish the degree of environmental temporal autocorrelation in our data, we calculated the Pearson correlation coefficient, using the *cor.test* function, between the different individually mean-centred weather variables and their values from 5 previous days. Additionally, we tested the correlation between individually mean-centred torpor use between time t and t-1. The correlation coefficients revealed that there were varying degrees of temporal autocorrelation present in the different environmental variables (Table S5.2). Moon disk illumination showed a strong temporal trend, which is to be expected as this variable is the equivalent to the changing moon phases. Barometric pressure showed high correlation between conditions at time t and time t-1 (correlation = 0.70) and some correlation between t and t-2 (correlation = 0.36), showing a cyclic pattern across the 5 previous days (Fig. S5.4). Humidity showed correlation between conditions at time t and time t-1 (correlation = 0.56) and also revealed a cyclic pattern across 5 consecutive days (Fig. S5.4). T_a_, precipitation, wind and ΔBP all showed non-cyclic patterns and low levels of correlation in conditions comparing time t and up to 5 days earlier (correlation < 0.37). Torpor use on a given night was not strongly correlated with torpor use expressed on the previous night (correlation = 0.30, Fig. S5.5).

**Table S5.1**: Pearson correlation coefficient values between individually mean-centred conditions on a given night (t) and conditions from 5 previous nights (t-1 to t-5).

|  | Ta | Rain | Wind | Humidity | BP | ΔBP | Moon | Torpor |
| --- | --- | --- | --- | --- | --- | --- | --- | --- |
| *t-1* | 0.37 | 0.02 | 0.10 | 0.56 | 0.70 | 0.18 | 0.82 | 0.30 |
| *t-2* | 0.16 | -0.23 | 0.02 | 0.15 | 0.36 | -0.24 | 0.69 | - |
| *t-3* | -0.08 | -0.21 | -0.04 | -0.09 | 0.18 | -0.21 | 0.58 | - |
| *t-4* | 0.03 | -0.12 | -0.17 | -0.23 | 0.10 | -0.10 | 0.47 | - |
| *t-5* | 0.05 | -0.12 | -0.04 | -0.31 | 0.01 | 0.03 | 0.34 | - |


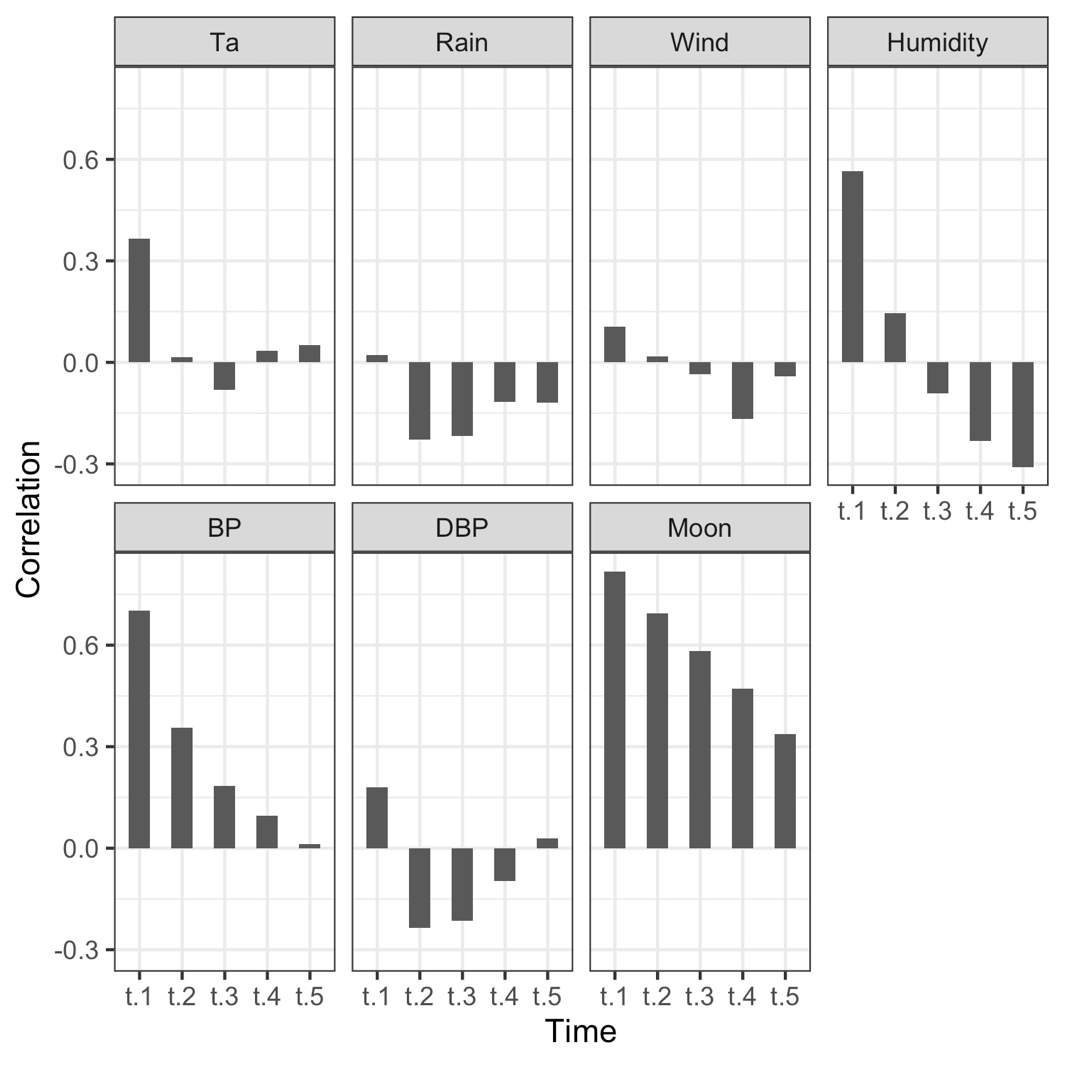


**Figure S5.3**: Correlation coefficients from Table S4.1, illustrating the temporal autocorrelation for each of the environmental variables for up to 5 days prior to the current conditions.


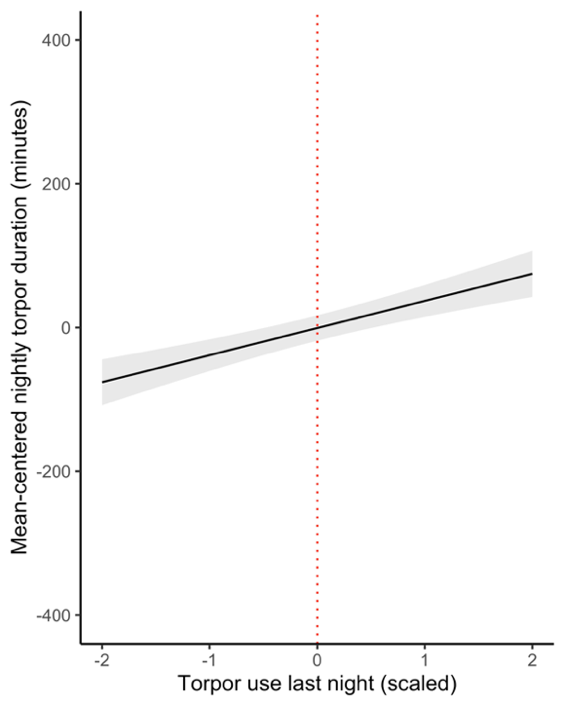


**Figure S5.4:** The effect of torpor use the night before (i.e. t-1, scaled) on the mean-centred torpor use on a current night when accounting for effects from the best within-subject model. The y-axis is fitted to the same range as Fig. 3 in Results to allow for direct comparisons with environmental effects at time t.
